# Definitions, measurement, and reporting of progression-free survival in randomized clinical trials and observational studies of patients with advanced non-small-cell lung cancer treated with immunotherapy: a scoping review

**DOI:** 10.1016/j.esmorw.2025.100118

**Published:** 2025-03-05

**Authors:** M.V. Verschueren, V.P. Tassopoulou, R. Visscher, J. Schuurkamp, B.J.M. Peters, M. Koopman, E.M.W. van de Garde, A.C.G. Egberts, L.T. Bloem

**Affiliations:** 1 Department of Clinical Pharmacy, St. Antonius Hospital, Utrecht/Nieuwegein, The Netherlands; 2 Division of Pharmacoepidemiology and Clinical Pharmacology, Utrecht Institute for Pharmaceutical Sciences, Utrecht University, Utrecht, The Netherlands; 3 Department of Medical Oncology, University Medical Centre Utrecht, Utrecht University, Utrecht, The Netherlands; 4 Department of Clinical Pharmacy, University Medical Centre Utrecht, Utrecht, The Netherlands

**Keywords:** progression-free survival, observational studies, randomized clinical trials, advanced non-small-cell lung cancer, immunotherapy, scoping review

## Abstract

**Background:**

Evidence from observational studies is increasingly used in oncology to complement evidence from clinical trials. Commonly used endpoints to evaluate oncology medicines are overall survival (OS) and progression-free survival (PFS). However, comparing PFS across observational studies and with clinical trials can be challenging due to differences in its definition and measurement. This scoping review investigated how PFS was defined, measured, and reported in randomized clinical trials (RCTs) and observational studies of patients with advanced non-small-cell lung cancer (NSCLC) treated with immunotherapy.

**Materials and methods:**

This scoping review included RCTs and observational studies that measured PFS in advanced NSCLC patients treated with immunotherapy. ASReview, an open-source artificial intelligence-assisted tool, was used to screen and prioritize relevant studies from records identified from PubMed and Embase between 2012 and 2023. Information on study characteristics, PFS definitions, and measurements was extracted.

**Results:**

Forty RCTs and 144 observational studies were included. Most RCTs were conducted across multiple continents (70%), while most observational studies were conducted in Asia (62%). In contrast to RCTs, many observational studies lacked reporting on the end date of PFS measurement (69%), the type of radiological imaging (59%), and the imaging reviewer (78%). For observational studies that did report on PFS definitions and measurements, these often differed from those in RCTs, particularly regarding event definitions, the start and stop dates for PFS measurement, and tumor assessment schedules.

**Conclusions:**

In contrast to RCTs, observational studies often lack reporting on PFS definitions and measurements, and if reported, they differ across observational studies and between them and RCTs. Since observational studies are important for complementing evidence, aligning PFS definition and measurement criteria with those used in RCTs, along with detailed reporting, is needed. However, some variability in PFS measurement characteristics is unavoidable, and therefore, PFS estimates from observational studies should be interpreted critically and carefully.

## Highlights

• Comparing PFS across observational studies (OBSs) and with RCTs is challenging.
• We examine how PFS is defined, measured, and reported in RCTs and OBSs.
• In contrast to RCTs, OBSs often lack reporting on PFS definitions and measurements.
• If reported, the PFS definitions and measurements differ across OBSs and between RCTs.
• Aligning PFS characteristics from OBSs with RCTs and detailed reporting are needed.

## Introduction

Evidence from observational studies is increasingly used to complement evidence from clinical trials to understand the benefits and risks of new oncology medicines because the generalizability of these trials to daily clinical practice is often limited.^1^ Patients in randomized clinical trials (RCTs) typically constitute a highly selective sample, are often younger, fitter, and with fewer comorbidities than the broader patient population treated in daily clinical practice. In addition, the variability in treatment adherence and outcome assessments is much greater in clinical practice compared with the strictly defined and controlled conditions of RCTs.^2^^,^^3^

An important aspect when using evidence from observational studies to complement that from RCTs is the definition, measurement, and reporting of endpoints. Over the past decade, the primary outcome in evaluating oncology medicines has shifted from overall survival (OS) to progression-free survival (PFS), since PFS is not influenced by subsequent treatment and requires shorter follow-up periods than OS.^4^ However, PFS is subject to inconsistencies in its definition and measurement, among others, due to the use of varying criteria [e.g. Response Evaluation Criteria in Solid Tumors (RECIST), immune-RECIST (iRECIST), or clinical criteria] and varying response assessment intervals.^5^, ^6^, ^7^, ^8^ These inconsistencies may lead to estimates of PFS that are not comparable, either within or between studies. For example, if one treatment arm is assessed more frequently than another within the same study, disease progression might be detected earlier, leading to biased absolute and relative PFS estimates. This issue extends to comparisons between studies, especially between observational studies and RCTs, with their often inherent differences in data collection.

These issues underscore the need for standardized reporting for observational studies to enhance the interpretability and reliability of PFS outcomes. In addition to existing general guidelines on reporting standards for observational studies,^9^^,^^10^ the European Society for Medical Oncology (ESMO) and the American Society of Clinical Oncology (ASCO) have recently introduced specific guidance to oncology research.^11^^,^^12^ Despite these developments, the degree of variation in how PFS is defined, measured, and reported across different study types remains unclear. Therefore, this scoping review aimed to evaluate how PFS was defined, measured, and reported for RCTs and observational studies of patients with unresectable advanced non-small-cell lung cancer (NSCLC) treated with immunotherapy. The population of unresectable advanced NSCLC was chosen due to its relatively short PFS and the use of immunotherapy as a standard treatment option.

## Materials and methods

This scoping review was conducted in accordance with the Joanna Briggs Institute manual for scoping reviews^13^ and the checklist of Preferred Reporting Items for Systematic Reviews and Meta-analyses—Extension for Scoping Review (PRISMA-ScR).^14^

### Eligibility criteria

This scoping review aimed to include all scientific publications of phase III RCTs and observational studies of patients with advanced unresectable NSCLC that compared the PFS of at least one programmed cell death protein 1 (PD-1)/programmed death-ligand 1 (PD-L1) or cytotoxic T-lymphocyte antigen 4 (CTLA-4) inhibiting immunotherapy treatment with one or more other treatments. For observational studies, this also included comparisons with data from clinical trials, either with trial estimates or with individual patient data. Phase I or II trials, interim analyses, updates, subgroup analyses, or publications not written in English were excluded.

### Information sources, search strategy, and study selection

A systematic search for available literature was carried out in the following two databases: PubMed and Embase. The databases were searched for articles published between January 2012 and March 2023. The full search term for each database is provided in Supplementary Table S1, available at https://doi.org/10.1016/j.esmorw.2025.100118.

The retrieved study records were first transferred to Rayyan QCRI and deduplicated. Then, the remaining records were transferred to an artificial intelligence (AI)-assisted review tool named ASReview. This open-source software systematically screens records with researcher-trained and continuously updated machine-learning algorithms.^15^ For this study, we used naïve Bayes for classification and term frequency–inverse document frequency for feature extraction. Two reviewers (MVV and RV) independently reviewed the titles and abstracts prioritized by ASReview to identify relevant reports. The reviewers stopped the title and abstract screening when the stopping rule of a minimum of 2250 records and 25 subsequent irrelevant records was reached.^16^ For both reviewers, the stopping rule was met after screening 2250 records, and the remaining records were labeled irrelevant by the review tool without interference from the reviewers. Both authors independently read the full text of the selected reports. Disagreements about inclusion were resolved by discussion with a third reviewer (LTB).

### Data extraction

Two reviewers (MVV and VT) independently extracted data from the included studies using the data extraction form provided in Supplementary Table S2, available at https://doi.org/10.1016/j.esmorw.2025.100118. Disagreements were resolved by discussion until consensus was reached, or with an additional reviewer (LTB).

The following characteristics were extracted: main author, year of publication, publication journal, initiated by pharmaceutical industry, continent, site type (multicenter or single center), type of data, type of comparison (number of treatment arms for RCTs and number of contemporary or historical cohorts for observational studies), follow-up time for the total study population, stage of disease, histology, line of treatment, type of treatment of interest (any type of PD-1/PD-L1 or CTLA-4 inhibitor alone, or in combination with chemotherapy, targeted agents, or other immunotherapy), cycle duration for the treatment of interest, follow-up time for the treatment of interest, comparator treatment (any type of systemic treatment or trial estimate or individual patient data trial), cycle duration for the comparator treatment, follow-up time for the comparator treatment. The following characteristics of PFS measurement were extracted: progression-defining events, start and end dates used for PFS calculation, radiological and/or non-radiological criteria (and if radiological, the type of criteria), type of radiological imaging, radiological imaging reviewer, and tumor assessment schedule. The type of radiological criteria is further categorized based on conventional RECIST v1.1 introduced in 2009,^17^ the newer response criteria (immune-related,^18^ immune,^19^ and immune-modified RECIST,^20^ respectively, introduced in 2013, 2017, and 2018) and a category of other or modified criteria. The characteristic ‘response assessment schedule’ was categorized into highly detailed, moderately detailed, and not detailed, as described in Supplementary Table S3, available at https://doi.org/10.1016/j.esmorw.2025.100118.

### Data synthesis

Descriptive statistics were used to summarize and compare the findings between RCTs and observational studies. The completeness of PFS definitions and measurements reported for each observational study was assessed using a scoring system with 1 point for each characteristic of PFS measurement, leading to a maximum total score of 8 points. For each observational study characteristic, the distribution of reporting was presented in a boxplot.

## Results

### Search strategy and study selection

**Figure 1:**
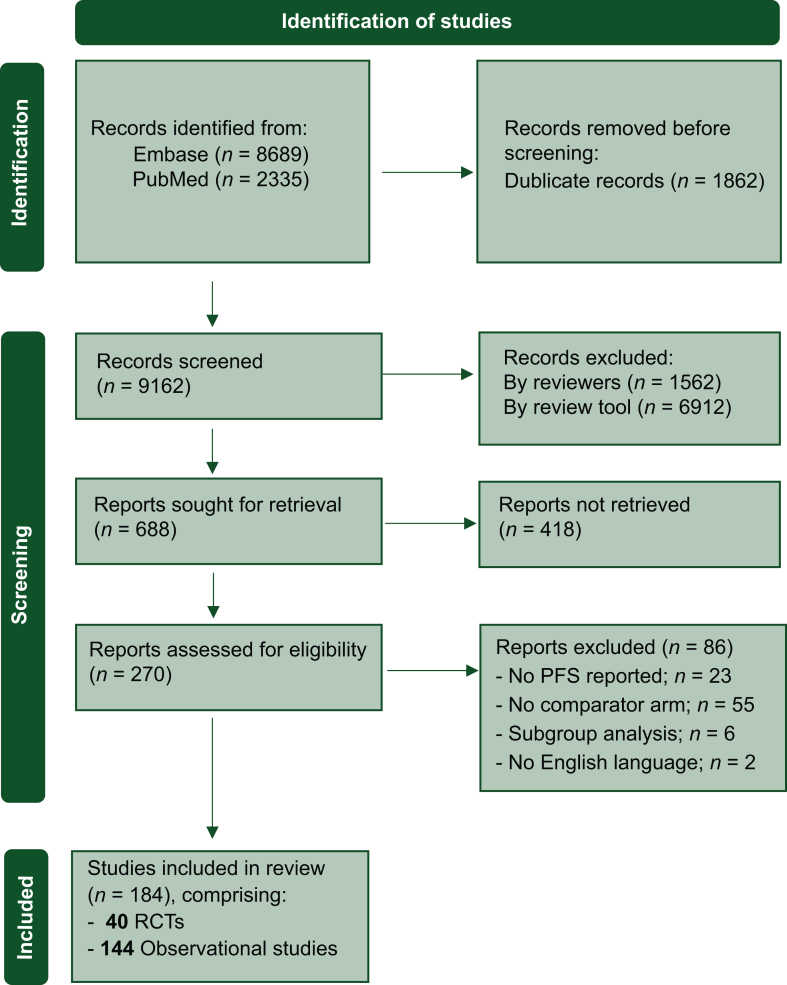
**PRISMA—flow diagram.** PRISMA, Preferred Reporting Items for Systematic Reviews and Meta-analyses; RCT, randomized clinical trial.

The initial search yielded a total of 9162 records after deduplication. Of these, 8474 were excluded based on title and abstract screening using the AI-assisted review tool ASReview. Once the stopping rule was met, 688 reports were sought for retrieval; however, 418 could not be retrieved, mainly due to their status as conference abstract from Embase. After assessing 270 full-text reports for eligibility, 86 more reports were excluded. A total of 184 studies were thus included for data extraction. Of these, 40 were RCTs (Refs. 1-40 in the Supplementary Material, available at https://doi.org/10.1016/j.esmorw.2025.100118) and 144 were observational studies (Refs. 41-184 in the Supplementary Material, available at https://doi.org/10.1016/j.esmorw.2025.100118) (Figure 1).

### Study characteristics

Most included RCTs were published between 2021 and 2023 (48%) in journals with a median impact factor of 45.9 [interquartile range (IQR) 20.4-70.7] (Table 1). All RCTs were initiated by the pharmaceutical industry, and were mostly conducted across multiple continents (70%). Most RCTs included patient populations with only stage IV disease (50%), with both non-squamous and squamous histologies (58%), and receiving first-line treatment (80%). In addition, most RCTs compared two treatment arms (90%), with immunotherapy most often as the treatment of interest (38%) and chemotherapy most often as the comparator (62%).

**Table 1:** Characteristics of the included clinical trials and observational studies

Characteristics	RCTs	Observational studies
	*N* = 40	*N* = 144
Study information		
Year of publication, *n* (%)		
2015-2017	7 (18)	1 (1)
2018-2020	14 (35)	43 (30)
2021-2023	19 (48)	100 (69)
Journal impact factor		
Median (IQR)	45.9 (20.4-70.7)	4.4 (2.9-5.3)
Not reported	0	1 (1)
Study design		
Initiated by pharmaceutical industry, *n* (%)		
Yes	40 (100)	5 (3)
No	0 (0)	139 (97)
Continent, *n* (%)		
Asia	12 (30)	90 (62)
America	0 (0)	36 (25)
Europe	0 (0)	12 (8)
Australia	0 (0)	3 (3)
Multicontinent	28 (70)	1 (1)
Not reported	0 (0)	2 (1)
Site type, *n* (%)		
Multicenter	40 (100)	53 (37)
Single center	0 (0)	89 (62)
Not reported	0 (0)	2 (1)
Type of data, *n* (%)		
Electronic health records	0 (0)	74 (51)
EHR combined with other data	0 (0)	13 (9)
Registry/database	0 (0)	11 (8)^a^
Clinical trial	40 (100)	0 (0)
Other	0 (0)	2 (1)^b^
Not reported	0 (0)	44 (31)
Type of comparison, *n* (%)		
For trials		
Two treatment arms	36 (90)	NA
More than two treatment arms	4 (10)	NA
For observational studies		
One cohort versus trial estimate (descriptive)	NA	34 (24)
One cohort versus IPD trial	NA	4 (3)
Two cohorts: contemporary	NA	82 (57)
Two cohorts: historical	NA	8 (6)
More than two cohorts: contemporary	NA	16 (11)
Follow-up time in months		
Total study population, median (IQR)	11.7 (9.0-16.3)	13.9 (9.3-20.2)
Not reported, *n* (%)	20 (45)	63 (44)
Stage of disease, *n* (%)		
Stage III^c^	2 (5)	15 (10)
Stage IV	20 (50)	72 (52)
Stage III^c^ and IV	18 (45)	40 (28)
Not reported	0 (0)	17 (12)
Histology, *n* (%)		
Non-squamous	7 (18)	47 (33)
Squamous	10 (7)	2 (1)
Both	23 (58)	91 (63)
Not reported	0 (0)	4 (3)
Line of treatment, *n* (%)		
1L	32 (80)	36 (25)
2L or later lines	8 (20)	52 (36)
Different lines	0 (0)	42 (29)
Not reported	0 (0)	14 (10)
Treatment of interest		
Type of treatment, *n* (% is *n*/*N*^d^)		
Immunotherapy (monotherapy)	17 (38)	48 (30)
Immunotherapy + chemotherapy	15 (33)	42 (26)
Immunotherapy + immunotherapy (+ chemotherapy)	9 (20)	0 (0)
Immunotherapy + (chemo)radiotherapy	1 (2)	22 (14)
Immunotherapy + anti-angiogenesis (+ chemotherapy)	3 (7)	22 (14)
Other	0 (0)	1 (1)^e^
Two or more of the above treatments within one arm	0 (0)	26 (16)
Cycle duration, *n* (% = *n*/*N*^d^)		
2 weeks	6 (13)	23 (14)
3 weeks	29 (64)	28 (18)
Other	9 (20)	24 (15)
Not reported	0 (0)	85 (53)
Follow-up time in months		
Median (IQR)	14.7 (12.2-18.9)	14.3 (9.2-18.6)
Not reported, *n* (% is *n*/*N*^d^)	20 (45)	135 (84)
Comparator treatment		
Type of treatment, *n* (% = *n*/*N*^d^)		
Immunotherapy (monotherapy)	2 (5)	48 (32)
Immunotherapy + chemotherapy	0 (0)	3 (2)
Anti-angiogenesis (+chemotherapy or immunotherapy)	1 (3)	15 (10)
Chemotherapy	25 (62)	23 (15)
(chemo)radiotherapy (+immunotherapy)	0 (0)	14 (9)
Placebo (+combinations)	12 (30)	0 (0)
Two or more of the above treatments within one arm	0 (0)	9 (6)
NA (comparison with trial)	NA	38 (25)
Cycle duration, *n* (% = *n*/*N*^d^)		
2 weeks	2 (5)	3 (2)
3 weeks	32 (80)	29 (19)
Other	5 (15)	12 (8)
Not reported	0 (0)	106 (71)
Follow-up time in months		
Median (IQR)	14.6 (10.9-17.8)	16.5 (12.8-29.5)
Not reported, *n* (% is *n*/*N*^f^)	32 (71)	127 (85)

1L, first-line treatment; 2L, second-line treatment; IPD, individual patient data; IQR, interquartile range; NA, not applicable.

a One study used interviews and the other study used data reported by physicians.

b Seven studies used disease-specific registries and four studies did not specify the type of registry used.

c Only studies in patients with unresectable stage III disease were included.

d The total number of treatment arms defined as treatment of interest was *n* = 45 for clinical trials and *n* = 161 for observational studies.

e Immunotherapy + anti-osteoporosis.

f The total number of treatment arms defined as comparator treatment; this percentage was *n* = 40 for clinical trials and *n* = 150 for observational studies.

Most observational studies were published between 2021 and 2023 (69%) in journals with a median impact factor of 4.4 (IQR 2.9-5.3). Most observational studies were not initiated by the pharmaceutical industry (97%), and were conducted in Asia (62%), in single centers (62%), and used electronic health records (51%). Most observational studies included patient populations with stage IV disease (54%), with both non-squamous and squamous histologies (63%), and receiving second-line or higher-line treatment (36%). In addition, most observational studies compared treatments between two contemporary cohorts (57%), with immunotherapy most often as the treatment of interest (30%) and as the comparator treatment (32%).

The treatments in the RCTs with two treatment arms and the observational studies with two cohorts are presented in a circle plot, respectively, in Supplementary Figures S1 and S2, available at https://doi.org/10.1016/j.esmorw.2025.100118. RCTs most often compared immunotherapy monotherapy with chemotherapy, while observational studies most often compared immunochemotherapy with immunotherapy monotherapy.

### PFS measurement characteristics

**Figure 2:**
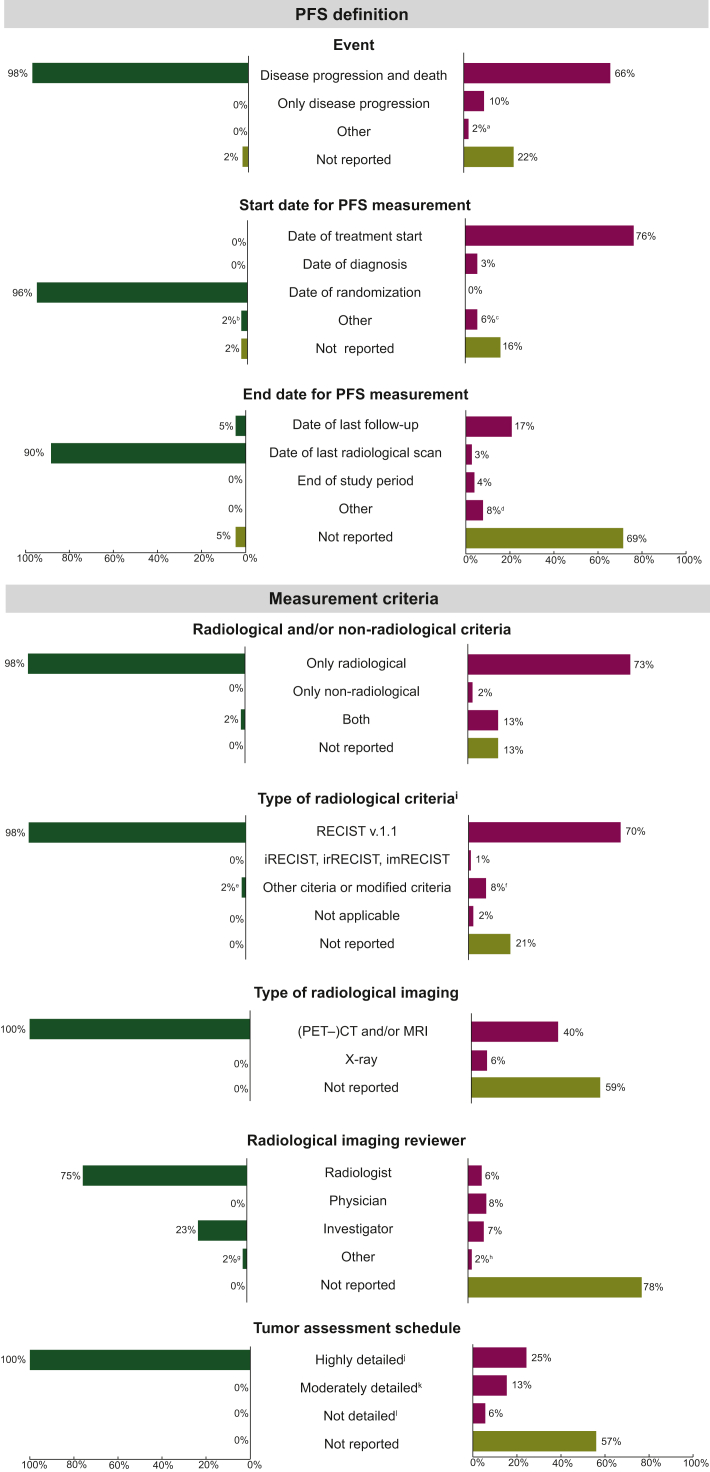
**Horizontal bar chart displaying PFS measurement characteristics of the included clinical trials and observational studies.** (PET–)CT, (positron emission tomography–)computed tomography; imRECIST, immune-modified RECIST; irRECIST, immune-related RECIST; iRECIST, immune RECIST; MRI, magnetic resonance imaging; mWHO, modified World Health Organization; PFS, progression-free survival; RANO, Response Assessment in Neuro-Oncology; RECIST, response evaluation criteria of solid tumors. ^a^Three observational studies used other event definitions: disease progression, death, or change of treatment (*n* = 1), disease progression or change of treatment (*n* = 1), and tumor relapse or death (*n* = 1). ^b^One clinical trial used the date of sub-study registration as start date of PFS evaluation. ^c^Nine observational studies used other start dates for PFS evaluation: the last date of (chemo)radiotherapy (*n* = 7), the last date of radiotherapy plus extra days to correct for immortal time bias (*n* = 1), and the first day of no evidence of disease progression (*n* = 1). ^d^Nine observational studies used other end dates for PFS evaluation: the last contact date or cut-off date if earlier (*n* = 6), and the last contact date of start date of new treatment line (*n* = 1). ^e^One clinical trial used the mWHO criteria to detect disease progression. ^f^Eleven observational studies used other or modified radiological criteria: RECIST modified to detect pseudoprogression (*n* = 4), modified RECIST (*n* = 5), immune-related response criteria (*n* = 1), and RANO criteria (*n* = 1). ^g^In one clinical trial, radiological imaging was reviewed by a combination of a qualified study physician and a radiologist to review radiological imaging. ^h^Four observational studies used other radiological imaging reviewers: a combination of a radiologist and a physician (*n* = 2), and a combination of an investigator and a radiologist (*n* = 1). ^i^Percentages are calculated by dividing the number of each category of ‘type of radiological criteria’ by the total number of studies that used radiological criteria. ^j^Highly detailed response assessment schedules are expressed in exact units such as cycles, days, weeks, or months with no significant allowance for variation (±7 days or less). For example, response assessments occur every 6 weeks. ^k^Moderately detailed response assessment schedules are expressed in units such as cycles, days, weeks, or months with a significant allowance for variation (>7 days). For example, response assessments occur approximately every 2-3 months. ^l^Not detailed response assessment schedules lacking precise timeframes. For example, response assessments occur at the physician’s discretion.

All PFS measurement characteristics are presented in Figure 2. For most RCTs, PFS was defined with events including both progression and death (98%), and follow-up from the date of randomization (96%) to the date of the last radiological scan (90%). For measurement criteria, radiological criteria solely were used for most RCTs (98%), with RECIST v.1.1 used in 98% of studies. Disease progression was assessed using (positron emission tomography–)computed tomography [(PET–)CT] and/or magnetic resonance imaging (MRI) for all RCTs and radiological images were reviewed by a radiologist for 75% of RCTs. The response assessment schedule was highly detailed for all RCTs. In addition, the reporting rate for RCTs was high, with fewer than 5% of measurement characteristics not reported.

Reporting for observational studies was low, with significant proportions of studies not providing details on key PFS measurement characteristics: event definition (22%), start date (16%), end date (69%), type of radiological imaging (59%), imaging reviewer (78%), and tumor assessment schedule (57%). When reported, 66% of observational studies defined PFS based on both progression and death events. For the follow-up period, 76% of studies used the date of treatment initiation and 17% used the date of last follow-up. Regarding measurement criteria, only radiological criteria were used in 73% of the studies, only non-radiological criteria in 2%, and a combination of both in 13%. Among studies using radiological criteria, 70% employed RECIST v1.1. Disease progression was measured using (PET–)CT and/or MRI for 75% of observational studies and radiological images were reviewed by different reviewers. The level of detail for the tumor assessment schedule varied from highly detailed (25%), moderately detailed (13%), to not detailed (6%).

For all observational studies, the completeness of reporting on PFS definitions and measurement characteristics did not vary according to study information and study design characteristics (Supplementary Table S4, available at https://doi.org/10.1016/j.esmorw.2025.100118).

## Discussion

This study was the first to systematically evaluate how PFS was defined, measured, and reported across RCTs and observational studies. The results show that, in contrast to RCTs, observational studies often lack reporting on PFS definitions and measurements, with 69% not reporting the end date for PFS measurement, 59% not reporting the type of radiological imaging, 78% not reporting the imaging reviewer, and 57% not reporting the tumor assessment schedule. For studies that did report on PFS definitions and measurements, these often differed from those in RCTs, particularly regarding event definitions, the start and stop dates for PFS measurement, and tumor assessment schedules. These findings hinder the interpretability and reliability of PFS outcomes of observational studies, limiting their ability to complement evidence from RCTs.

The low level of reporting of PFS measurement characteristics in observational studies included lacking information on the end date of PFS calculation, the type of radiological imaging and its reviewer, and the tumor assessment schedule. This lack of information can lead to unreliable estimates of PFS that are difficult to interpret and unreliable to use in clinical practice, undermining clinicians’ ability to make informed treatment choices. At the time these studies were conducted, general reporting standards for observational research, such as STROBE^9^ and RECORD-PE,^10^ were available and included recommendations for clearly defining outcomes. However, specific guidance to oncological observational studies, such as those later provided by the ESMO^11^ and ASCO,^13^ was not available, which may have contributed to the insufficient reporting. Although the introduction of these guidance documents is a step forward, they still do not provide specific instructions on reporting PFS measurement characteristics. Detailed reporting instructions on definitions of PFS, including events and follow-up times, and measurement criteria for PFS, including radiological criteria, assessment intervals, radiological imaging, and reviewers, are needed to improve the interpretability and reliability of PFS estimates from observational studies.

The variability in PFS definitions can be attributed to both fundamental flaws in study design choices and inherent limitations in the design of observational studies. One such fundamental flaw is using only disease progression as relevant event in PFS estimates, which could lead to biased estimates of PFS. An inherent limitation of observational studies is that PFS is measured from the date of treatment start, whereas in RCTs PFS is measured from the date of randomization. Also, the observed variability in PFS measurement criteria could result from inherent limitations of the observational studies since they leverage data from real-world clinical practices rather than highly standardized settings. For example, response assessments in observational studies occur less frequently and consistently than in RCTs, causing delayed or missed detection of disease progression, introducing assessment bias in PFS comparisons with RCTs as well as other observational studies.^5^^,^^21^ While some of the variabilities in PFS measurement criteria, such as the choice of imaging modalities or the frequency of response assessments, may be unavoidable due to the practical constraints of routine clinical care, others can be minimized through high-quality study design and methodologies. A recommended approach is to use the target trial emulation framework, which helps to design observational studies by closely replicating the design of hypothetical RCTs.^22^ Aligning key elements of trials, including strict PFS definitions, radiological criteria, imaging reviewers, and—where feasible—assessment schedules, reduces the variability in PFS measurement characteristics between observational studies and between observational studies and RCTs, thereby reducing bias in PFS comparisons between studies.

Some inherent variability in PFS measurement characteristics between observational studies and RCTs will always remain, which limits the ability to use evidence from observational studies to complement evidence from RCTs. In clinical practice, physicians evaluate disease progression to guide individual treatment decisions, resulting in less standardized measurement of PFS compared with RCTs. In clinical practice, the evaluation of disease progression is also based on clinical criteria such as worsening of clinical symptoms and performance status rather than solely on RECIST-evaluated radiological imaging.^23^ Additionally, the timing and use of scans in clinical practice are based on clinical needs and available resources rather than strict predefined protocols. In contrast, RCTs only use RECIST-evaluated radiological imaging and strict assessment schedules to evaluate disease progression. These variations in evaluation methods can result in biased PFS outcomes.^5^^,^^24^ This can be illustrated by the recent findings from the PACIFIC-R study,^25^ an observational study designed to complement the PACIFIC RCT^26^ by evaluating the effectiveness of adjuvant durvalumab in patients with unresectable NSCLC. The PACIFIC-R study reported a longer median PFS compared with the PACIFIC RCT (24.9 versus 16.9 months). Similarly, findings from our previous study on durvalumab showed longer PFS in patients treated in clinical practice compared with those in the PACIFIC RCT (27.1 versus 16.9 months).^27^ Both examples illustrate that without a standardized approach, PFS from observational studies may not capture the same clinical outcome as PFS from RCTs, raising the question whether PFS outcomes in observational studies can be reliably used to complement PFS outcomes from RCTs. Using the term ‘real-world PFS’ for PFS outcomes in observational studies may help to highlight the difference in measurement and context, as also discussed by others.^28^^,^^29^ Other endpoints, such as time to treatment discontinuation and time to treatment failure, have been proposed as more pragmatic alternatives for assessing treatment effects in clinical practice, although they also have their limitations.^30^ Nevertheless, PFS estimates from observational studies are valuable in situations where there is a total lack of evidence from RCTs. For example, they provide valuable insights into specific subpopulations not represented in RCTs and into treatment comparisons where no head-to-head RCT is available. While PFS estimates from observational studies are fit for purpose in these situations, they should be evaluated critically due to the inherent variability in their measurement.

Several limitations of our scoping review should be considered. Firstly, the review was limited in scope as it focused primarily on immunotherapy treatments for advanced NSCLC, which may not fully represent the broader range of issues across different types of cancer and treatment modalities. However, we believe that the observed variation in PFS measurement characteristics and lack of reporting are likely to apply to other settings as well. Additionally, while there is a small risk of missing studies due to the AI-assisted review tool, the review of nearly 10 000 studies supports the reliability of our findings, even if a few studies were missed.

### Conclusion

The findings from our scoping review show that, in contrast to RCTs, observational studies often lack reporting on PFS definitions and measurements. When these characteristics are reported, they often differ across observational studies and between them and RCTs. Since observational studies are important for complementing evidence, aligning PFS definition and measurement criteria with those used in RCT, along with detailed reporting on these aspects, is needed. However, some variability in PFS measurement characteristics is unavoidable, and therefore, PFS estimates from observational studies should be interpreted critically and carefully.
