# Supplementary material for: Definitions, measurement, and reporting of progression-free survival in randomized clinical trials and observational studies of patients with advanced non-small-cell lung cancer treated with immunotherapy: a scoping review
Source: ESMO Real World Data Digit Oncol. 2025 Mar 5;7:100118. doi: 10.1016/j.esmorw.2025.100118 (PMC12836502; doi:10.1016/j.esmorw.2025.100118)
Supplement: Supplementary Figure S1 [file mmc1.pdf]

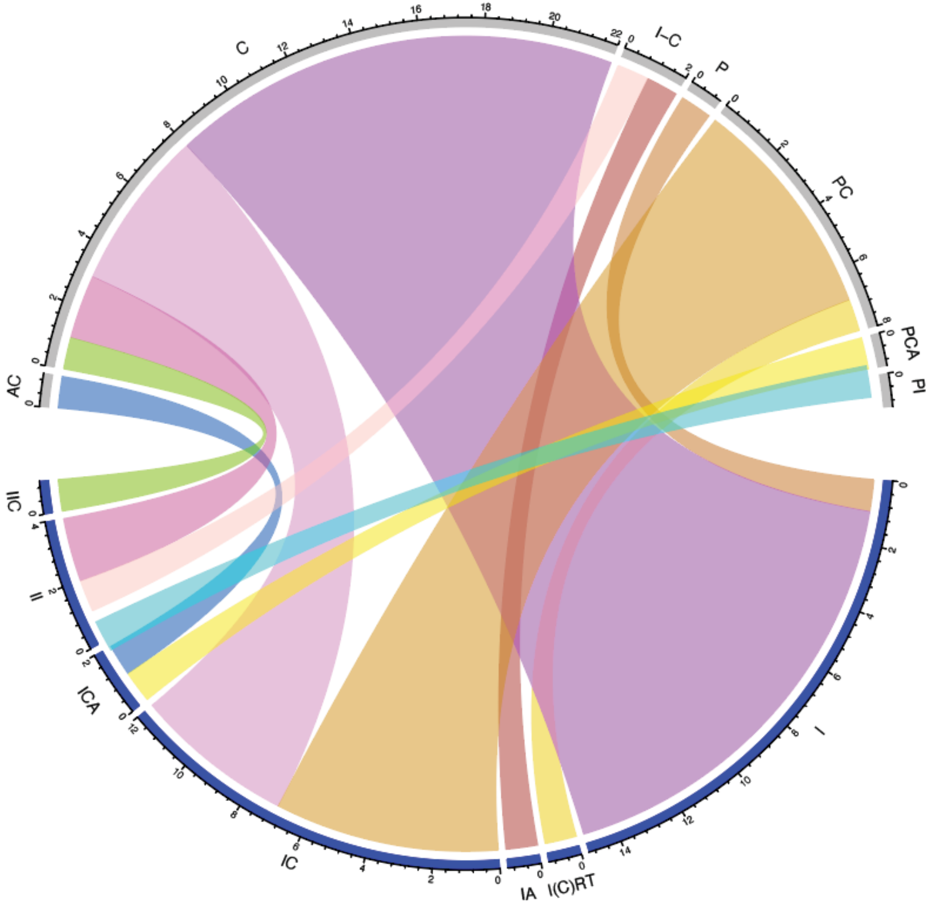

Figure S1: Circle plot of treatment comparisons in clinical trial with two treatment arms (n = 36). The segment borders are blue for the treatment of interest and grey for the comparator treatment.
Abbreviations: **AC**, anti-angiogenesis + chemotherapy; **C**, chemotherapy; **I**¸ immunotherapy; **IC**, immunotherapy + chemotherapy; **ICA**, immunotherapy + chemotherapy + anti-angiogenesis; **I(C)RT**, Immunotherapy + (chemo)radio-therapy; **II**, Immunotherapy + immunotherapy; **IIC**, Immunotherapy + immunotherapy + chemotherapy; **P**, placebo; **PC**, placebo + chemotherapy; **PI**; Placebo + immunotherapy; **PCA** , placebo + chemotherapy + anti-angiogenesis.
