# Supplementary material for: Definitions, measurement, and reporting of progression-free survival in randomized clinical trials and observational studies of patients with advanced non-small-cell lung cancer treated with immunotherapy: a scoping review
Source: ESMO Real World Data Digit Oncol. 2025 Mar 5;7:100118. doi: 10.1016/j.esmorw.2025.100118 (PMC12836502; doi:10.1016/j.esmorw.2025.100118)
Supplement: Supplementary Figure S2 [file mmc2.pdf]

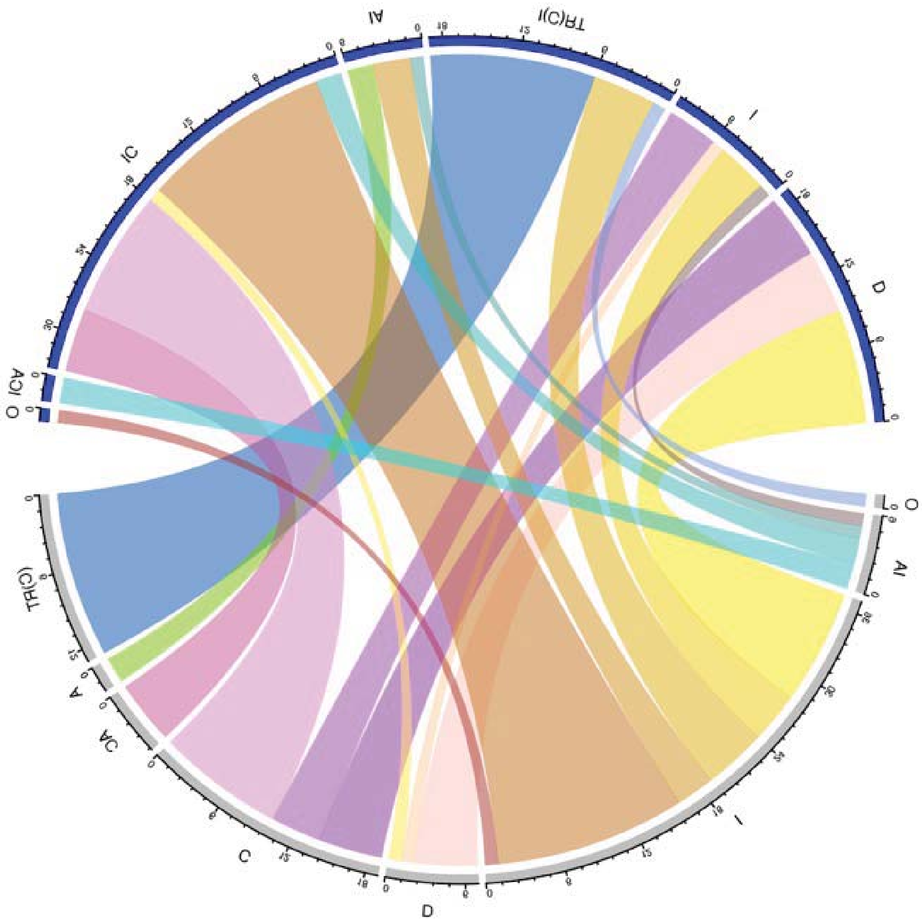

Figure S2: Circle plot of treatment comparisons in observational studies with two treatment cohorts (n = 90). The segment borders are blue for the treatment of interest and grey for the comparator treatment.
Abbreviations: **A**, anti-angiogenesis; **AC**, anti-angiogenesis + chemotherapy; **C**, chemotherapy; **D**, different treatments per arm; **ICA**, immunotherapy + chemotherapy + anti-angiogenesis; **I** , immunotherapy (monotherapy); **IA**, immunotherapy + anti-angiogenesis; **IC**, immunotherapy + chemotherapy; **I(C)RT**, immunotherapy + (chemo)radiotherapy; **O**, other.
