# Supplementary material for: Definitions, measurement, and reporting of progression-free survival in randomized clinical trials and observational studies of patients with advanced non-small-cell lung cancer treated with immunotherapy: a scoping review
Source: ESMO Real World Data Digit Oncol. 2025 Mar 5;7:100118. doi: 10.1016/j.esmorw.2025.100118 (PMC12836502; doi:10.1016/j.esmorw.2025.100118)
Supplement: Supplementary Data [file mmc3.docx]

**Supplementary files**

Table S1. Search strategy in Pubmed and Embase

| **Database** | **Search strategy** |
| --- | --- |
| PubMed | "Programmed Cell Death 1 Receptor"[Mesh] OR "programmed cell death 1 receptor" [tiab] OR "programmed cell death 1 receptors" [tiab] OR “Programmed Cell Death Protein 1 Inhibitor” [tiab] OR “Programmed Cell Death Protein 1 Inhibitors” [tiab] OR "pd-1 inhibitor" [tiab] OR "pd-1 inhibitors" [tiab] OR "PD-L1 inhibitor"[tiab] OR “PD 1 inhibitor” [tiab] OR “PD 1 inhibitors” [tiab] OR "PD-L1 inhibitors"[tiab] OR “Programmed Death-Ligand 1 Inhibitors” [tiab] OR “Programmed Death Ligand 1 Inhibitors” [tiab] OR “Programmed Death-Ligand 1 Inhibitor” [tiab] OR “Programmed Death Ligand 1 Inhibitor” [tiab] OR "CTLA-4 Antigen"[Mesh] OR "ctla-4 antigen" [tiab] OR "cytotoxic Tlymphocyte antigen 4" [tiab] OR “CTLA 4 inhibitors” [tiab] OR “CTLA 4 Inhibitors” [tiab] OR “CTLA- 4 Inhibitor” [tiab] OR “CTLA 4 Inhibitor” [tiab] OR “Cytotoxic T-Lymphocyte-Associated Protein 4 Inhibitors” [tiab] OR “Cytotoxic T Lymphocyte Associated Protein 4 Inhibitors” [tiab] OR “Cytotoxic T-Lymphocyte-Associated Protein 4 Inhibitor” [tiab] OR “Cytotoxic T Lymphocyte Associated Protein  4 Inhibitor” [tiab] OR "anti-pd-1" [tiab] OR "anti-pd-l1" [tiab] OR "anti-ctla-4" [tiab] OR "immune checkpoint inhibitor" [tiab] OR "immune checkpoint inhibitors" [tiab] OR “immune checkpoint blocker” [tiab] OR “immune checkpoint blockers” [tiab] OR "ICI" [tiab] OR "ici therapy" [tiab] OR "pembrolizumab" [tiab] OR "nivolumab"[tiab] OR "atezolizumab"[tiab] OR "durvalumab" [tiab] OR "ipilimumab" [tiab] OR "avelumab" [tiab] OR "tremelimumab" [tiab] OR "cemiplimab" [tiab] OR “Antibodies, Monoclonal, Humanized” [tiab]  AND  "Carcinoma, Non-Small-cell lung"[Mesh] OR NSCLC* [tiab] OR "non-small cell lung cancer*" [tiab] OR "non-small cell lung carcinoma*" [tiab] OR “Carcinoma, Non Small Cell Lung” [tiab] OR “Carcinomas, Non-Small-Cell Lung” [tiab] OR “Lung Carcinoma, Non-Small-Cell” [tiab] OR “Lung Carcinomas, Non-Small-Cell” [tiab] OR “Non-Small-Cell Lung Carcinomas” [tiab] OR “Nonsmall Cell Lung Cancer” [tiab] OR “Carcinoma, Non-Small Cell Lung” [tiab]  AND  "Progression-free survival"[Mesh] OR "Progression free survival" [tiab] OR "progression-free survival" [tiab] OR PFS [tiab] OR "progression-free" [tiab] OR "progression free" [tiab] OR "time to progression"[tiab] OR "Survival, Progression-Free" [tiab]” |
| Embase | "programmed death 1 receptor”/exp OR “cytotoxic T lymphocyte antigen 4”/exp OR 'programmed cell death 1 receptor':ab,ti OR 'programmed cell death 1 receptors':ab,ti OR 'programmed cell death protein 1 inhibitor':ab,ti OR 'programmed cell death protein 1 inhibitors':ab,ti OR 'pd-l1 inhibitor':ab,ti OR 'pd 1 inhibitor':ab,ti OR 'pd 1 inhibitors':ab,ti OR 'pd-l1 inhibitors':ab,ti OR 'programmed death ligand 1 inhibitors':ab,ti OR 'programmed death ligand 1 inhibitor':ab,ti OR 'ctla 4 antigen':ab,ti OR 'cytotoxic t-lymphocyte antigen 4':ab,ti OR 'ctla 4 inhibitors':ab,ti OR 'ctla 4 inhibitor':ab,ti OR 'cytotoxic t lymphocyte associated protein 4 inhibitors':ab,ti OR 'cytotoxic t lymphocyte associated protein 4 inhibitor':ab,ti OR 'anti-pd-1':ab,ti OR 'anti-pd-l1':ab,ti OR 'anti-ctla-4':ab,ti OR 'immune checkpoint inhibitor':ab,ti OR 'immune checkpoint inhibitors':ab,ti OR 'immune checkpoint blocker':ab,ti OR 'immune checkpoint blockers':ab,ti OR 'ici':ab,ti OR 'ici therapy':ab,ti OR 'pembrolizumab':ab,ti OR 'nivolumab':ab,ti OR 'atezolizumab':ab,ti OR 'durvalumab':ab,ti OR 'ipilimumab':ab,ti OR 'avelumab':ab,ti OR 'tremelimumab':ab,ti OR 'cemiplimab':ab,ti OR 'antibodies monoclonal humanized':ab,ti OR 'immunotherapy':ab,ti  AND  ‘non small cell lung cancer’/exp OR 'nsclc*':ab,ti OR 'non small cell lung cancer*':ab,ti OR 'non small cell lung carcinoma*':ab,ti OR 'carcinomas non small cell lung':ab,ti OR 'lung carcinoma non small cell':ab,ti OR 'lung carcinomas non small cell':ab,ti OR 'non-small-cell lung carcinomas':ab,ti OR 'nonsmall cell lung cancer':ab,ti OR 'carcinoma non small cell lung':ab,ti  AND  ‘progression free survival’/exp OR 'progression free survival':ab,ti OR 'pfs':ab,ti OR 'progression-free':ab,ti OR 'time to progression':ab,ti OR 'survival progression free':ab,ti  AND  [2011-2023]/py |

Table S2. Data extraction template

|  | Specification |
| --- | --- |
| Name study | … |
| Number in search file | … |
| First author | … |
| Publication journal | … |
| Publication year | … |
| Journal impact factor | … |
| Initiated by pharmaceutical industry | Yes/no |
| Continent | America/Africa/Asia/Oceania/Multi-continent/Not reported |
| Type of study design | RCT/observational study |
| Type of site | Multicenter / Single center / other |
|  | If multicenter, how many centers involved: … |
| Type of data | EHR/EHR data supplemented with other data/ Registries (treatment, disease or other, please specify)/ Claims data/ Clinical trial data/ Other, please specify /Not reported |
| Type of comparison | For RCTs;  - two treatment arms  - >two treatment arms  For observational studies  - one cohort versus trial estimate  - one cohort versus IPD trial  - two cohorts with contemporary treatments  - two cohorts: one with treatment of interest and one historical cohort  - > two cohorts (contemporary or historical) |
| Follow-up time (in months) | - For the total study population  - For the treatment of interest  - For the comparator treatment |
| Stage of disease | Stage IIIb/ Stage IIIc/ Stage IVa / Stage IVb / Other, please specify / Not reported |
| Type of histology | Squamous / Adeno / Large cell / Not other specified / Other, please specify / Not reported |
| Line of treatment | 1L / 2L/ higher lines/ Other, please specify / Not reported |
| Type of treatment of interest | Any PD-1/L1 or CTLA-4 inhibitor alone or in combination with other treatments. Specify all anticancer drugs used within one treatment regimen.  … |
| Type of comparator | Specify all comparators (any type of systemic treatment or trial estimates or IPD trial)  … |
| Cycle duration (in weeks) | - For the treatment of interest  …  - For the comparator treatment  … |
| Event definition | Disease progression and death / Disease progression alone / Other, please specify/ Not reported |
| Start date for calculation PFS | Date of treatment start / Date of diagnoses / Date of randomization / Other, please specify / Not reported |
| End date for PFS calculation | Date of last follow-up / Date of last radiological scan / End of study period / Other, please specify / Not reported |
| Radiological and/or non-radiological criteria | Only radiological / Only non-radiological / Both / Not Reported |
|  | If non-radiological criteria were used, please specify |
| Type of radiological criteria | RECIST v1.1 / iRECIST / irRECIST / imRECIST / modified criteria, please specify modifications / other criteria, please specify / Not reported |
| Type of radiological imaging | (PET-)CT / MRI / X-ray / Other, please specify / Not reported |
| Radiological imaging reviewer | Radiologist / Physician / Investigator / Other, please specify / Not reported |
| Response assessment schedule | … |

Table S3. Categorization of ‘response assessment schedule’.

| **Category** | **Definition** | **Example** |
| --- | --- | --- |
| Highly detailed | Response assessment schedules that are expressed in exact unite such as cycles, days, weeks, months, with no significant allowance for variation (+/- 7 days) | *‘Response assessment occur every 6 weeks’* |
| Moderately detailed | Response assessment schedules that are expressed in exact unite such as cycles, days, weeks, months, with allowance for variation (> 7 days) | ‘*Response assessments occur every 2 to 3 months’* |
| Not detailed | Response assessments schedules without precise timeframes | ‘*Response assessments occur at the physician’s discretion’* |
| Not reported | - | - |

Table S4. Completeness of reporting of PFS evaluation characteristics for each characteristics of observational studies.

| **Study characteristics** | | **Observational studies (n = 144)** | **Completeness of reporting of PFS evaluation characteristics*** |
| --- | --- | --- | --- |
|  |  | N (%) |  |
| Year of publication | 2015-2017 | 1 (1) | NA |
|  | 2018-2020 | 43 (30) | 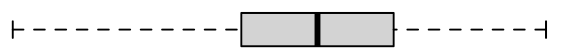 |
|  | 2021-2023 | 100 (69) | 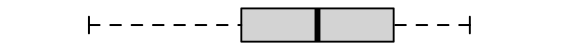 |
| Journal impact factor | < 4.4 | 72 (50) | 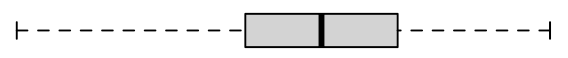 |
|  | ≥ 4.4 | 72 (50) | 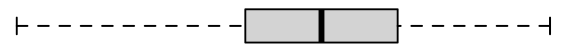 |
| Sponsored by the industry | Yes | 5 (3) | 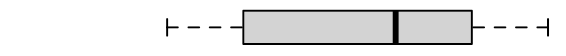 |
|  | No | 139 (97) | 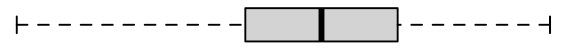 |
| Continent | Asia | 90 (62) | 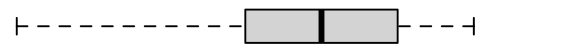 |
|  | America | 12 (8) | **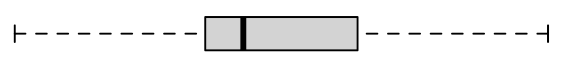** |
|  | Europe | 36 (25) | 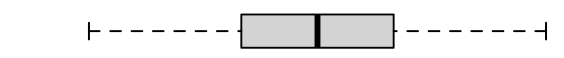 |
|  | Australia | 3 (3) | **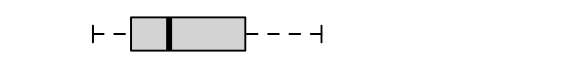** |
|  | Multicontinent | 1(1) | NA |
|  | Not reported | 2 (1) | NA |
| Site type | Multicenter | 53 (37) | 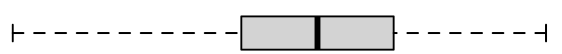 |
|  | Single center | 89 (62) | 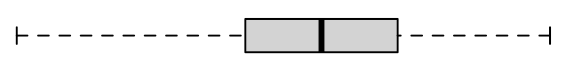 |
|  | Not reported | 2 (1) | NA |
| Type of data | EHR | 74 (51) | 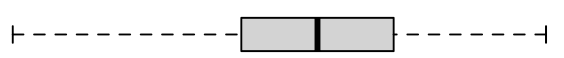 |
|  | EHR combined with other data | 13 (9) | 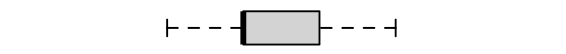 |
|  | Registry/database | 11 (8) | 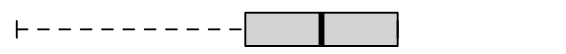 |
|  | Other | 2 (1) | NA |
|  | Not reported | 44 (31) | **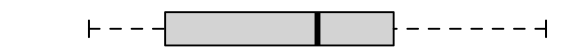** |
| Type of comparison | One cohort with trial (descriptive) | 34 (24) | 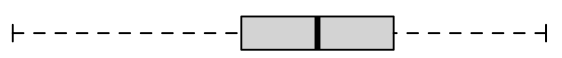 |
|  | One cohort: Benchmark trial (statistical) | 4 (3) | 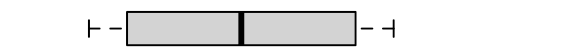 |
|  | Two cohort: Contemporary | 82 (57) | 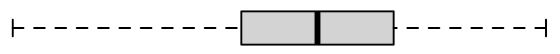 |
|  | Two cohort: Historical | 8 (6) | 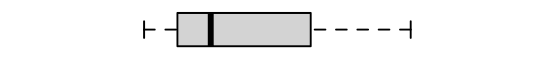 |
|  | >Two cohort: Contemporary | 16 (11) | 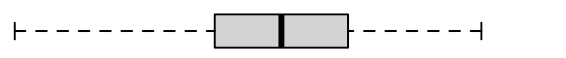 |
| Follow-up time | Reported | 81 (56) | 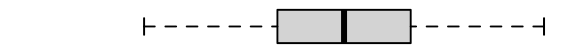 |
|  | Not reported | 63 (44) | **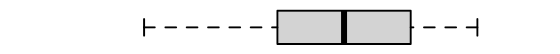** |
| Stage of disease | Stage III | 15 (10) | 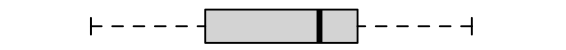 |
|  | Stage IV | 72 (52) | 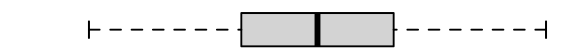 |
|  | Both | 40 (28) | 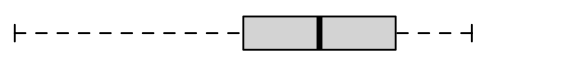 |
|  | Not reported | 17 (12) | 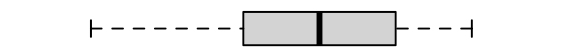 |
| Histology | Non-squamous | 47 (33) | 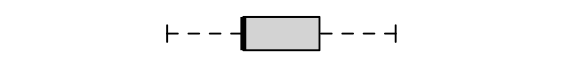 |
|  | Squamous | 2 (1) | NA |
|  | Both | 91 (63) | 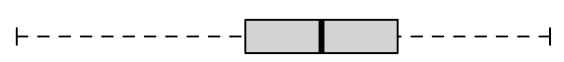 |
|  | Not reported | 4 (3) | 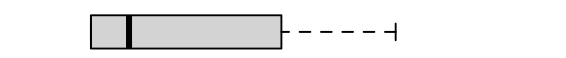 |
| Line of treatment | 1L | 36 (25) | 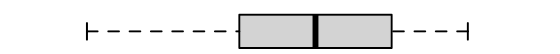 |
|  | 2L or later lines | 52 (36) | 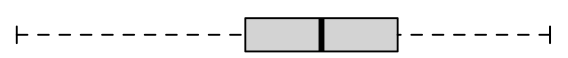 |
|  | Different lines | 42 (29) | 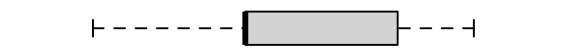 |
|  | Not reported | 14 (10) | 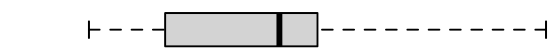 |
|  |  | | **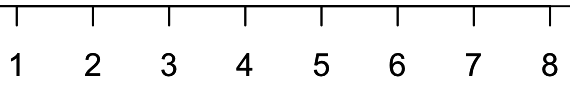**  Total score |

Abbreviations: EHR, electronic health records; NA, not applicable.

* For each study, the completeness of reporting for all PFS evaluation characteristics was assessed using a scoring system with a total score of 8. Each missing variable resulted in a reduction of 1 point from the total score.

**References**

**RCTs**

1. Antonia SJ, Villegas A, Daniel D, Vicente D, Murakami S, Hui R, et al. Durvalumab after Chemoradiotherapy in Stage III Non–Small-Cell Lung Cancer. New England Journal of Medicine. 2017 Nov 16;377(20):1919–29.
2. Barlesi F, Vansteenkiste J, Spigel D, Ishii H, Garassino M, de Marinis F, et al. Avelumab versus docetaxel in patients with platinum-treated advanced non-small-cell lung cancer (JAVELIN Lung 200): an open-label, randomised, phase 3 study. Lancet Oncol. 2018 Nov 1;19(11):1468–79.
3. Brahmer J, Reckamp KL, Baas P, Crinò L, Eberhardt WEE, Poddubskaya E, et al. Nivolumab versus Docetaxel in Advanced Squamous-Cell Non–Small-Cell Lung Cancer. New England Journal of Medicine. 2015 Jul 9;373(2):123–35.
4. Borghaei H, Paz-Ares L, Horn L, Spigel DR, Steins M, Ready NE, et al. Nivolumab versus Docetaxel in Advanced Nonsquamous Non–Small-Cell Lung Cancer. New England Journal of Medicine. 2015 Oct 22;373(17):1627–39.
5. Boyer M, N S MA, Rodríguez-Abreu D, Park K, Ho Lee D, Çiçin I, et al. Pembrolizumab Plus Ipilimumab or Placebo for Metastatic Non-Small-Cell Lung Cancer With PD-L1 Tumor Proportion Score ≥ 50%: Randomized, Double-Blind Phase III KEYNOTE-598 Study. J Clin Oncol.2021;39:2327–38.
6. Carbone DP, Reck M, Paz-Ares L, Creelan B, Horn L, Steins M, et al. First-Line Nivolumab in Stage IV or Recurrent Non–Small-Cell Lung Cancer. New England Journal of Medicine. 2017 Jun 22;376(25):2415–26.
7. de Castro G, Rizvi NA, Schmid P, Syrigos K, Martin C, Yamamoto N, et al. NEPTUNE: Phase 3 Study of First-Line Durvalumab Plus Tremelimumab in Patients With Metastatic NSCLC. Journal of Thoracic Oncology. 2023 Jan 1;18(1):106–19.
8. Gandhi L, Rodríguez-Abreu D, Gadgeel S, Esteban E, Felip E, De Angelis F, et al. Pembrolizumab plus Chemotherapy in Metastatic Non–Small-Cell Lung Cancer. New England Journal of Medicine. 2018 May 31;378(22):2078–92.
9. Gettinger SN, Redman MW, Bazhenova L, Hirsch FR, Mack PC, Schwartz LH, et al. Nivolumab plus Ipilimumab vs Nivolumab for Previously Treated Patients with Stage IV Squamous Cell Lung Cancer: The Lung-MAP S1400I Phase 3 Randomized Clinical Trial. JAMA Oncol. 2021 Sep 1;7(9):1368–77.
10. Gogishvili M, Melkadze T, Makharadze T, Giorgadze D, Dvorkin M, Penkov K, et al. Cemiplimab plus chemotherapy versus chemotherapy alone in non-small cell lung cancer: a randomized, controlled, double-blind phase 3 trial. Nat Med. 2022 Nov 1;28(11):2374–80.
11. Govindan R, Szczesna A, Ahn MJ, Schneider CP, Fernando Gonzalez Mella P, Barlesi F, et al. JOURNAL OF CLINICAL ONCOLOGY Phase III Trial of Ipilimumab Combined With Paclitaxel and Carboplatin in Advanced Squamous Non-Small-Cell Lung Cancer. J Clin Oncol. 2017;35:3449–57.
12. Johnson ML, Chul Cho B, Luft A, Alatorre-Alexander J, Lucien Geater S, Laktionov K, et al. Durvalumab With or Without Tremelimumab in Combination With Chemotherapy as First-Line Therapy for Metastatic Non-Small-Cell Lung Cancer: The Phase III POSEIDON Study. J Clin Oncol. 2022;41:1213–27.
13. Hellmann MD, Ciuleanu TE, Pluzanski A, Lee JS, Otterson GA, Audigier-Valette C, et al. Nivolumab plus Ipilimumab in Lung Cancer with a High Tumor Mutational Burden. New England Journal of Medicine. 2018 May 31;378(22):2093–104.
14. Herbst RS, Giaccone G, de Marinis F, Reinmuth N, Vergnenegre A, Barrios CH, et al. Atezolizumab for First-Line Treatment of PD-L1–Selected Patients with NSCLC. New England Journal of Medicine. 2020 Oct 1;383(14):1328–39.
15. Jotte R, Cappuzzo F, Vynnychenko I, Stroyakovskiy D, Rodríguez-Abreu D, Hussein M, et al. Atezolizumab in Combination With Carboplatin and Nab-Paclitaxel in Advanced Squamous NSCLC (IMpower131): Results From a Randomized Phase III Trial. Journal of Thoracic Oncology. 2020 Aug 1;15(8):1351–60.
16. Nishio M, Barlesi F, West H, Ball S, Bordoni R, Cobo M, et al. Atezolizumab Plus Chemotherapy for First-Line Treatment of Nonsquamous NSCLC: Results From the Randomized Phase 3 IMpower132 Trial. Journal of Thoracic Oncology. 2021 Apr 1;16(4):653–64.
17. Paz-Ares L, Ciuleanu TE, Cobo M, Schenker M, Zurawski B, Menezes J, et al. First-line nivolumab plus ipilimumab combined with two cycles of chemotherapy in patients with non-small-cell lung cancer (CheckMate 9LA): an international, randomised, open-label, phase 3 trial. Lancet Oncol. 2021 Feb 1;22(2):198–211.
18. Paz-Ares L, Vicente D, Tafreshi A, Robinson A, Soto Parra H, Mazières J, et al. A Randomized, Placebo-Controlled Trial of Pembrolizumab Plus Chemotherapy in Patients With Metastatic Squamous NSCLC: Protocol-Specified Final Analysis of KEYNOTE-407. Journal of Thoracic Oncology. 2020;15(10):1657–69.
19. Peters S, Dziadziuszko R, Morabito A, Felip E, Gadgeel SM, Cheema P, et al. Atezolizumab versus chemotherapy in advanced or metastatic NSCLC with high blood-based tumor mutational burden: primary analysis of BFAST cohort C randomized phase 3 trial. Nat Med. 2022 Sep 1;28(9):1831–9.
20. Planchard D, Reinmuth N, Orlov S, Fischer JR, Sugawara S, Mandziuk S, et al. ARCTIC: durvalumab with or without tremelimumab as third-line or later treatment of metastatic non-small-cell lung cancer. Annals of Oncology. 2020 May 1;31(5):609–18.
21. Mok TSK, Wu YL, Kudaba I, Kowalski DM, Cho BC, Turna HZ, et al. Pembrolizumab versus chemotherapy for previously untreated, PD-L1-expressing, locally advanced or metastatic non-small-cell lung cancer (KEYNOTE-042): a randomized, open-label, controlled, phase 3 trial. The Lancet. 2019 May 4;393(10183):1819–30.
22. Reck M, Barlesi F, Yang JCH, Westeel V, Felip E, Özgüroğlu M, et al. Avelumab Versus Platinum-Based Doublet Chemotherapy as First-Line Treatment for Patients With High-Expression Programmed Death-Ligand 1–Positive Metastatic NSCLC: Primary Analysis From the Phase 3 JAVELIN Lung 100 Trial. Journal of Thoracic Oncology. 2023;
23. Reck M, Rodríguez-Abreu D, Robinson AG, Hui R, Csőszi T, Fülöp A, et al. Pembrolizumab versus Chemotherapy for PD-L1–Positive Non–Small-Cell Lung Cancer. New England Journal of Medicine. 2016 Nov 10;375(19):1823–33.
24. Rittmeyer A, Barlesi F, Waterkamp D, Park K, Ciardiello F, von Pawel J, et al. Atezolizumab versus docetaxel in patients with previously treated non-small-cell lung cancer (OAK): a phase 3, open-label, multicentre randomised controlled trial. The Lancet. 2017 Jan 21;389(10066):255–65.
25. Rizvi NA, Cho BC, Reinmuth N, Lee KH, Luft A, Ahn MJ, et al. Durvalumab with or Without Tremelimumab vs Standard Chemotherapy in First-line Treatment of Metastatic Non-Small Cell Lung Cancer: The MYSTIC Phase 3 Randomized Clinical Trial. JAMA Oncol. 2020 May 1;6(5):661–74.
26. Sezer A, Kilickap S, Gümüş M, Bondarenko I, Özgüroğlu M, Gogishvili M, et al. Cemiplimab monotherapy for first-line treatment of advanced non-small-cell lung cancer with PD-L1 of at least 50%: a multicentre, open-label, global, phase 3, randomised, controlled trial. 2021. 397.
27. Shi Y, Wu L, Yu X, Xing P, Wang Y, Zhou J, et al. Sintilimab versus docetaxel as second-line treatment in advanced or metastatic squamous non-small-cell lung cancer: an open-label, randomized controlled phase 3 trial (ORIENT-3). Cancer Commun. 2022 Dec 1;42(12):1314–30.
28. Socinski MA, Jotte RM, Cappuzzo F, Orlandi F, Stroyakovskiy D, Nogami N, et al. Atezolizumab for First-Line Treatment of Metastatic Nonsquamous NSCLC. New England Journal of Medicine. 2018 Jun 14;378(24):2288–301
29. Sugawara S, Lee JS, Kang JH, Kim HR, Inui N, Hida T, et al. Nivolumab with carboplatin, paclitaxel, and bevacizumab for first-line treatment of advanced nonsquamous non-small-cell lung cancer. Annals of Oncology. 2021 Sep 1;32(9):1137–47.
30. Wang J, Lu S, Yu X, Hu Y, Sun Y, Wang Z, et al. Tislelizumab plus chemotherapy vs chemotherapy alone as first-line treatment for advanced squamous non-small-cell lung cancer a phase 3 randomized clinical trial. JAMA Oncol. 2021 May 1;7(5):709–17.
31. Wang Z, Wu L, Li B, Cheng Y, Li X, Wang X, et al. Toripalimab Plus Chemotherapy for Patients With Treatment-Naive Advanced Non-Small-Cell Lung Cancer: A Multicenter Randomized Phase III Trial (CHOICE-01). J Clin Oncol. 2022;41:651–63.
32. West H, McCleod M, Hussein M, Morabito A, Rittmeyer A, Conter HJ, et al. Atezolizumab in combination with carboplatin plus nab-paclitaxel chemotherapy compared with chemotherapy alone as first-line treatment for metastatic non-squamous non-small-cell lung cancer (IMpower130): a multicentre, randomised, open-label, phase 3 trial. Lancet Oncol. 2019 Jul 1;20(7):924–37.
33. Wu YL, Lu S, Cheng Y, Zhou C, Wang J, Mok T, et al. Nivolumab Versus Docetaxel in a Predominantly Chinese Patient Population With Previously Treated Advanced NSCLC: CheckMate 078 Randomized Phase III Clinical Trial. Journal of Thoracic Oncology. 2019 May 1;14(5):867–75.
34. Yang JCH, Han B, De La Mora Jiménez E, Lee JS, Koralewski P, Karadurmus N, et al. Pembrolizumab With or Without Lenvatinib for First-Line Metastatic Non‒Small-Cell Lung Cancer With PD-L1 Tumor Proportion Score ≥1% (LEAP-007): A Randomized, Double-Blind, Phase 3 Trial. Journal of Thoracic Oncology. 2023 Dec;
35. Wu YL, Lu S, Cheng Y, Zhou C, Wang J, Mok T, et al. Nivolumab Versus Docetaxel in Predominantly Chinese Patient Population With Previously Treated Advanced NSCLC: CheckMate 078 Randomized Phase III Clinical Trial. Journal of Thoracic Oncology. 2019 May 1;14(5):867–75.
36. Zhang L, Wang Z, Fang J, Yu Q, Han B, Cang S, et al. Final overall survival data of sintilimab plus pemetrexed and platinum as First-Line treatment for locally advanced or metastatic nonsquamous NSCLC in the Phase 3 ORIENT-11 study. Lung Cancer. 2022 Sep 1;171:56–60.
37. Zhou C, Chen G, Huang Y, Zhou J, Lin LZ, Feng J, et al. Camrelizumab plus carboplatin and pemetrexed versus chemotherapy alone in chemotherapy-naive patients with advanced non-squamous non-small-cell lung cancer (CameL): a randomised, open-label, multicentre, phase 3 trial. Lancet Respir Med. 2021 Mar 1;9(3):305–14.
38. Zhou Q, Chen M, Jiang O, Pan Y, Hu D, Lin Q, et al. Sugemalimab versus placebo after concurrent or sequential chemoradiotherapy in patients with locally advanced, unresectable, stage III non-small-cell lung cancer in China (GEMSTONE-301): interim results of a randomised, double-blind, multicentre, phase 3 trial. Lancet Oncol. 2022 Feb 1;23(2):209–19.
39. Zhou C, Wang Z, Sun Y, Cao L, Ma Z, Wu R, et al. Sugemalimab versus placebo, in combination with platinum-based chemotherapy, as first-line treatment of metastatic non-small-cell lung cancer (GEMSTONE-302): interim and final analyses of a double-blind, randomised, phase 3 clinical trial. Lancet Oncol. 2022 Feb 1;23(2):220–33
40. Zhou C, Wu L, Fan Y, Wang Z, Liu L, Chen G, et al. Sintilimab Plus Platinum and Gemcitabine as First-Line Treatment for Advanced or Metastatic Squamous NSCLC: Results From a Randomized, Double-Blind, Phase 3 Trial (ORIENT-12). Journal of Thoracic Oncology. 2021 Sep 1;16(9):1501–11.

**Observational studies**

1. Abe T, Saito S, Iino M, Aoshika T, Ryuno Y, Ohta T, et al. Effect of durvalumab on local control after concurrent chemoradiotherapy for locally advanced non-small cell lung cancer in comparison with chemoradiotherapy alone. Thorac Cancer. 2021 Jan 1;12(2):245–50.
2. Abe T, Iino M, Saito S, Aoshika T, Ryuno Y, Ohta T, et al. Comparison of the Efficacy and Toxicity of Concurrent Chemoradiotherapy and Durvalumab and Concurrent Chemoradiotherapy Alone for Locally Advanced Non-small Cell Lung Cancer With N3 Lymph Node Metastasis. Anticancer Res. 2023 Feb 1;43(2):675–82.
3. Afzal MZ, Dragnev K, Shirai K. A tertiary care cancer center experience with carboplatin and pemetrexed in combination with pembrolizumab in comparison with carboplatin and pemetrexed alone in non-squamous non-small cell lung cancer. J Thorac Dis. 2018 Jun 1;10(6):3575–84.
4. Ahn BC, Pyo KH, Xin CF, Jung D, Shim HS, Lee CY, et al. Comprehensive analysis of the characteristics and treatment outcomes of patients with non-small cell lung cancer treated with anti-PD-1 therapy in real-world practice. J Cancer Res Clin Oncol. 2019;
5. Merino Almazán M, Duarte Pérez JM, Marín Pozo JF, Ortega Granados AL, Muros De Fuentes B, Quesada Sanz P, et al. A multicentre observational study of the effectiveness, safety and economic impact of nivolumab on non-small-cell lung cancer in real clinical practice. Int J Clin Pharm. 2019 Feb 15;41(1):272–9.
6. Alonso-García M, Sánchez-Gastaldo A, Muñoz-Fuentes MA, Molina-Pinelo S, Boyero L, Benedetti JC, et al. Real-World Analysis of Nivolumab and Atezolizumab Efficacy in Previously Treated Patients with Advanced Non-Small Cell Lung Cancer. Pharmaceuticals (Basel). 2022 May 1 [cited 2022 Jul 28];15(5).
7. Amrane K, Geier M, Corre R, Léna H, Léveiller G, Gadby F, et al. First-line pembrolizumab for non–small cell lung cancer patients with PD-L1 ≥50% in a multicenter real-life cohort: The PEMBREIZH study. Cancer Med. 2020 Apr 1;9(7):2309–16.
8. Attili I, Valenza C, Santoro C, Antonarelli G, Trillo Aliaga P, Del Signore E, et al. Comparison of real-world data (RWD) analysis on efficacy and post-progression outcomes with pembrolizumab plus chemo vs chemo alone in metastatic non-squamous non-small cell lung cancer with PD-L1 < 50%. Front Oncol. 2022 Aug 10;12.
9. Batra U, Chufal KS, Nathany S, Ahmad I, Chowdhary RL, Sharma M, et al. Immunotherapy in advanced non-small-cell lung cancer (NSCLC) after progression on chemotherapy: Real-world results from a prospective institutional cohort. Immunotherapy. 2022 Aug 1;14(11):851–8.
10. Bazhenova L, Kish J, Cai B, Caro N, Feinberg B. Real-world observational study of current treatment patterns and outcomes in recurrent or locally advanced/metastatic non-small cell lung cancer. Cancer Treat Res Commun. 2022 Jan 1;33.
11. Benjamin DJ, Chen S, Eldredge JB, Schokrpur S, Li D, Quan Z, et al. The Role of Chemotherapy Plus Immune Checkpoint Inhibitors in Oncogenic-Driven NSCLC: A University of California Lung Cancer Consortium Retrospective Study. JTO Clin Res Rep. 2022 Dec 1;3(12).
12. Bjørnhart B, Hansen KH, Jørgensen TL, Herrstedt J, Schytte T. Efficacy and safety of immune checkpoint inhibitors in a Danish real life non-small cell lung cancer population: a retrospective cohort study. Acta Oncol (Madr). 2019 Jul 3;58(7):953–61.
13. Bongiovanni A, Foca F, Menis J, Stucci SL, Artioli F, Guadalupi V, et al. Immune Checkpoint Inhibitors With or Without Bone-Targeted Therapy in NSCLC Patients With Bone Metastases and Prognostic Significance of Neutrophil-to-Lymphocyte Ratio. Front Immunol. 2021 Nov 10;12.
14. Bruni A, Scotti V, Borghetti P, Vagge S, Cozzi S, D’Angelo E, et al. A Real-World, Multicenter, Observational Retrospective Study of Durvalumab After Concomitant or Sequential Chemoradiation for Unresectable Stage III Non-Small Cell Lung Cancer. Front Oncol. 2021 Sep 28;11.
15. Chen Y, Wang Y, Yang Z, Hu M, Zhang Y, Qian F, et al. Pembrolizumab Alone or Combined With Chemotherapy in Advanced NSCLC With PD-L1 ≥50%: Results of a Retrospective Study. Front Oncol. 2021 Jun 28;11.
16. José ABS, Colomer-Aguilar C, Martínez-Caballero D, Massutí-Sureda B. Effectiveness and safety of atezolizumab, nivolumab and pembrolizumab in metastatic non-small cell lung cancer. In: Farmacia Hospitalaria. Grupo Aula Medica S.L.; 2021. p. 121–5.
17. Calpe-Armero P, Ferriols-Lisart R, Ferriols-Lisart F, Pérez-Pitarch A. Effectiveness of Nivolumab versus Docetaxel as Second-Line Treatment in Non-Small Cell Lung Cancer Patients in Clinical Practice. Chemotherapy. 2017 Nov 1;62(6):374–80.
18. Cavaille F, Peretti M, Garcia ME, Giorgi R, Ausias N, Vanelle P, et al. Real-world efficacy and safety of pembrolizumab in patients with non-small cell lung cancer: a retrospective observational study. Tumori. 2021 Feb 1;107(1):32–8.
19. Chen B, Wang J, Pu X, Li J, Wang Q, Liu L, et al. The efficacy and safety of immune checkpoint inhibitors combined with chemotherapy or anti-angiogenic therapy as a second-line or later treatment option for advanced non-small cell lung cancer: a retrospective comparative cohort study. Transl Lung Cancer Res. 2022 Oct 1;11(10):2111–24.
20. Chen Y, Kang S, Yan M. Atezolizumab plus carboplatin and nab-paclitaxel versus carboplatin and nab-paclitaxel as treatments for Chinese, treatment-naïve, stage IV, non-squamous, non-small-cell lung cancer patients: A retrospective analysis. Pharmacol Res Perspect. 2022 Jun 1;10(3).
21. Chen S, Wei H, Zhao W, Jiang W, Ning R, Zhou S, et al. PD-1/PD-L1 inhibitors plus anti-angiogenic agents with or without chemotherapy versus PD-1/PD-L1 inhibitors plus chemotherapy as second or later-line treatment for patients with advanced non-small cell lung cancer: A real-world retrospective cohort study. Front Immunol. 2022 Dec 7;13.
22. Chen M, Xu Y, Zhao J, Liu X, Liu X, Zhang D, et al. Comparison of Chemotherapy Plus Pembrolizumab vs. Chemotherapy Alone in EGFR-Mutant Non–small-Cell Lung Cancer Patients. Clin Lung Cancer. 2023 May 1;24(3):278–86.
23. Chen Y, Yang Z, Wang Y, Hu M, Zhang B, Zhang Y, et al. Pembrolizumab Plus Chemotherapy or Anlotinib vs. Pembrolizumab Alone in Patients With Previously Treated EGFR-Mutant NSCLC. Front Oncol. 2021 Apr 16;11.
24. Cheng Y, Yang B, Ouyang W, Jie C, Zhang W, Chen G, et al. Is ICI-based therapy better than chemotherapy for metastatic NSCLC patients who develop EGFR-TKI resistance? A real-world investigation. Front Oncol. 2022 Aug 23;12.
25. Chu X, Qiang H, Xie M, Li X, Zhao J, Wu Y, et al. Treatment efficacy of HER2-mutant lung adenocarcinoma by immune checkpoint inhibitors: a multicenter retrospective study. Cancer Immunology, Immunotherapy. 2022 Jul 1;71(7):1625–31.
26. Cramer-van der Welle CM, Verschueren M V., Tonn M, Peters BJM, Schramel FMNH, Klungel OH, et al. Real-world outcomes versus clinical trial results of immunotherapy in stage IV non-small cell lung cancer (NSCLC) in the Netherlands. Sci Rep. 2021 Dec 1;11(1).
27. Cui P, Li R, Huang Z, Wu Z, Tao H, Zhang S, et al. Comparative effectiveness of pembrolizumab vs. nivolumab in patients with recurrent or advanced NSCLC. Sci Rep. 2020 Dec 1;10(1).
28. Deng H, Lin X, Xie X, Yang Y, Wang L, Wu J, et al. Immune Checkpoint Inhibitors Plus Single-Agent Chemotherapy for Advanced Non-Small-Cell Lung Cancer After Resistance to EGFR-TKI. Front Oncol. 2021 Sep 20;11.
29. Descourt R, Greillier L, Perol M, Ricordel C, Auliac JB, Falchero L, et al. First-line single-agent pembrolizumab for PD-L1-positive (tumor proportion score ≥ 50%) advanced non-small cell lung cancer in the real world: impact in brain metastasis: a national French multicentric cohort (ESCKEYP GFPC study). Cancer Immunology, Immunotherapy. 2023 Jan 1;72(1):91–9.
30. Diker O, Olgun P. First-line pembrolizumab efficacy in patients with advanced non-small cell lung cancer: A Bi-center retrospective, real-life experience study. JBUON. 2021;26(3):844–52.
31. Dudnik E, Moskovitz M, Daher S, Shamai S, Hanovich E, Grubstein A, et al. Effectiveness and safety of nivolumab in advanced non-small cell lung cancer: The real-life data. Lung Cancer. 2018 Dec 1;126:217–23.
32. Faehling M, Schumann C, Christopoulos P, Hoffknecht P, Alt J, Horn M, et al. Durvalumab after definitive chemoradiotherapy in locally advanced unresectable non-small cell lung cancer (NSCLC): Real-world data on survival and safety from the German expanded-access program (EAP). Lung Cancer. 2020 Dec 1;150:114–22.
33. Feng Y, Tang L, Wang H, Liu Y, Yang S, Lin L, et al. Immune checkpoint inhibitors combined with angiogenic inhibitors in the treatment of locally advanced or metastatic lung adenocarcinoma patients. Cancer Immunology, Immunotherapy. 2023 Feb 1;72(2):449–59.
34. Figueiredo A, Almeida MA, Almodovar MT, Alves P, Araújo A, Araújo D, et al. Real-world data from the Portuguese Nivolumab Expanded Access Program (EAP) in previously treated Non Small Cell Lung Cancer (NSCLC). Pulmonology. 2020 Jan 1;26(1):10–7.
35. Fiorica F, Belluomini L, Stefanelli A, Santini A, Urbini B, Giorgi C, et al. Immune checkpoint inhibitor nivolumab and radiotherapy in pretreated lung cancer patients. American Journal of Clinical Oncology: Cancer Clinical Trials. 2018 Nov 1;41(11):1101–5.
36. Frost N, Kollmeier J, Misch D, Vollbrecht C, Grah C, Matthes B, et al. Pembrolizumab as First-Line Palliative Therapy in PD-L1 Overexpressing (≥ 50%) NSCLC: Real-world Results with Special Focus on PS ≥ 2, Brain Metastases, and Steroids. Clin Lung Cancer. 2021 Sep 1;22(5):411–22.
37. Fukui T, Okuma Y, Nakahara Y, Otani S, Igawa S, Katagiri M, et al. Activity of Nivolumab and Utility of Neutrophil-to-Lymphocyte Ratio as a Predictive Biomarker for Advanced Non–Small-Cell Lung Cancer: A Prospective Observational Study. Clin Lung Cancer. 2019 May 1;20(3):208-214.e2.
38. Geier M, Descourt R, Corre R, Léveiller G, Lamy R, Goarant É, et al. Real life second-line nivolumab in advanced non-small cell lung cancer: A French observational multicenter study of 259 patients (ABCT-IMMUNOBZH). Cancer Rep Rev. 2018;2(5).
39. Girard N, Smit HJM, Sibille A, et. al. PACIFIC-R real-world study: treatment duration and interim analysis of progression-free-survival in unresectable stage III NSCLC patients treated with durvalumab after chemoradiotherapy. 2021 p. Presented at: 2021 ESMO Congress; September 16-12.
40. Gong J, Gu J, Jiang L, Zhao D, Shao L, Chen X, et al. Clinical Study on the Efficacy of Bevacizumab in Combination with Pembrolizumab on Cellular Immune Function in the Treatment of Driver Gene-Negative Stage IV Lung Adenocarcinoma. J Oncol. 2022;2022.
41. Gu X, Shi Z, Shao L, Zhang Y, Zhang Y, Song Z, et al. Efficacy and safety of maintenance immune checkpoint inhibitors with or without pemetrexed in advanced non-squamous non-small cell lung cancer: a retrospective study. BMC Cancer. 2022 Dec 1;22(1).
42. Hong L, Lewis WE, Nilsson M, Patel S, Varghese S, Rivera MJ, et al. Limited Benefit from the Addition of Immunotherapy to Chemotherapy in TKI-Refractory EGFR-Mutant Lung Adenocarcinoma. Cancers (Basel). 2022 Jul 1;14(14).
43. Hsu JC, Lin JY, Hsu MY, Lin PC. Effectiveness and safety of immune checkpoint inhibitors: A retrospective study in Taiwan. PLoS One. 2018 Aug 1;13(8).
44. Hu R, Zhao Z, Shi Y, Shi M, Xia G, Yu S, et al. Immune checkpoint inhibitors combined with chemotherapy/ bevacizumab therapy for patients with advanced lung cancer and heavily treated with EGFR mutation: A retrospective analysis. J Thorac Dis. 2021 May 1;13(5):2959–67.
45. Hu J, Huang D, Wang Y, Li D, Yang X, Fu Y, et al. The efficacy of immune checkpoint inhibitors in advanced EGFR-Mutated non-small cell lung cancer after resistance to EGFR-TKIs: Real-World evidence from a multicenter retrospective study. Front Immunol. 2022 Sep 9;13.
46. Guberina M, Guberina N, Pöttgen C, Gauler T, Richlitzki C, Metzenmacher M, et al. Effectiveness of durvalumab consolidation in stage III non-small-cell lung cancer: focus on treatment selection and prognostic factors. Immunotherapy. 2022 Aug 1;14(12):927–44.
47. Guo X, Du H, Li J, Yang M, Xiong A, Zhang H, et al. Efficacy of ICIs on patients with oncogene-driven non-small cell lung cancer: a retrospective study. Cancer Drug Resistance. 2022;5(1):15–24.
48. He L, Chen X, Ding L, Zhang X. Clinical Efficacy of Antianlotinib Combined with Immune Checkpoint Inhibitors in the Treatment of Advanced Non-Small-Cell Lung Cancer and Its Effect on Serum VEGF, CEA, and SCC-Ag. J Oncol. 2022;2022.
49. Huang Y, Zhao JJ, Soon YY, Wong A, Aminkeng F, Ang Y, et al. Real-world experience of consolidation durvalumab after concurrent chemoradiotherapy in stage III non-small cell lung cancer. Thorac Cancer. 2022 Nov 1;13(22):3152–61.
50. Huang D, Cui P, Huang Z, Wu Z, Tao H, Zhang S, et al. Anti-PD-1/L1 plus anti-angiogenesis therapy as second-line or later treatment in advanced lung adenocarcinoma. J Cancer Res Clin Oncol. 2021 Mar 1;147(3):881–91.
51. Huang Z, Zhou C, Xiong Y, Yang F, Zeng F, Jiang W, et al. PD-1 inhibitor versus bevacizumab in combination with platinum-based chemotherapy for first-line treatment of advanced lung adenocarcinoma: A retrospective-real world study. Front Oncol. 2022 Nov 9;12.
52. Ikezawa Y, Mizugaki H, Morita R, Tateishi K, Yokoo K, Sumi T, et al. Current status of first-line treatment with pembrolizumab for non–small-cell lung cancer with high PD-L1 expression. Cancer Sci. 2022 Jun 1;113(6):2109–17.
53. Ismail RK, Schramel FMNH, van Dartel M, Pasmooij AMG, Cramer-van der Welle CM, Hilarius DL, et al. Individual patient data to allow a more elaborated comparison of trial results with real-world outcomes from first-line immunotherapy in NSCLC. BMC Med Res Methodol. 2023 Dec 1;23(1).
54. Ivanović M, Knez L, Herzog A, Kovačević M, Cufer T. Immunotherapy for Metastatic Non-Small Cell Lung Cancer: Real-World Data from an Academic Central and Eastern European Center. Oncologist. 2021 Dec 1;26(12):e2143–50.
55. Jung HA, Noh JM, Sun JM, Lee SH, Ahn JS, Ahn MJ, et al. Real world data of durvalumab consolidation after chemoradiotherapy in stage III non-small-cell lung cancer. Lung Cancer. 2020 Aug 1;146:23–9.
56. Kawachi H, Tamiya M, Taniguchi Y, Yokoyama T, Yokoe S, Oya Y, et al. Efficacy of Immune Checkpoint Inhibitor With or Without Chemotherapy for Nonsquamous NSCLC With Malignant Pleural Effusion: A Retrospective Multicenter Cohort Study. JTO Clin Res Rep. 2022 Jul 1;3(7).
57. Khan M, Zhao Z, Li X, Liao G. Anti-pd1 therapy plus whole-brain radiation therapy may prolong pfs in selected non–small cell lung cancer patients with brain metastases: A retrospective study. Int J Gen Med. 2021;14:8903–18.
58. Kim SH, Choi CM, Lee DH, Kim SW, Yoon S, Kim WS, et al. Clinical outcomes of nivolumab in patients with advanced non-small cell lung cancer in real-world practice, with an emphasis on hyper-progressive disease. J Cancer Res Clin Oncol. 2020 Nov 1;146(11):3025–36.
59. Kishi N, Matsuo Y, Shintani T, Ogura M, Mitsuyoshi T, Araki N, et al. Recurrence patterns and progression-free survival after chemoradiotherapy with or without consolidation durvalumab for stage III non-small cell lung cancer. J Radiat Res. 2023 Jan 20;64(1):142–53.
60. Kobayashi K, Nakachi I, Naoki K, Satomi R, Nakamura M, Inoue T, et al. Real-world Efficacy and Safety of Nivolumab for Advanced Non–Small-cell Lung Cancer: A Retrospective Multicenter Analysis. Clin Lung Cancer. 2018 May 1;19(3):e349–58.
61. Krefting F, Basara N, Schütte W, Späth-Schwalbe E, Alt J, Thiel S, et al. Clinical Experience of Immunotherapy Treatment: Efficacy and Toxicity Analysis of the Compassionate Use Program of Nivolumab in Patients with Advanced Squamous Cell Non-Small Cell Lung Cancer. Oncol Res Treat. 2019 May 1;42(5):243–54.
62. Kumar S, Joga S, Biswas B, Dabkara D, Prasad KT, Singh N, et al. Immune checkpoint inhibitors in advanced non–small cell lung cancer: A metacentric experience from India. Vol. 44, Current Problems in Cancer. Mosby Inc.; 2020.
63. Kuo CHS, Wang CC, Huang YC, Pavlidis S, Liu CY, Ko HW, et al. Comparison of a combination of chemotherapy and immune checkpoint inhibitors and immune checkpoint inhibitors alone for the treatment of advanced and metastatic non-small cell lung cancer. Thorac Cancer. 2019 May 1;10(5):1158–66.
64. Kurokawa K, Mitsuishi Y, Shimada N, Kawakami Y, Miura K, Miyawaki T, et al. Association between the efficacy and immune-related adverse events of pembrolizumab and chemotherapy in non-small cell lung cancer patients: a retrospective study. BMC Cancer. 2022 Dec 1;22(1).
65. Kwok WC, Cheong TF, Chiang KY, Ho JCM, Lam DCL, Ip MSM, et al. Clinical efficacy and safety of pemetrexed with or without either Bevacizumab or Pembrolizumab in patients with metastatic nonsquamous non–small cell carcinoma. Asia Pac J Clin Oncol. 2023 Feb 1;19(1):87–95.
66. Lang D, Huemer F, Rinnerthaler G, Horner A, Wass R, Brehm E, et al. Therapy Line and Associated Predictors of Response to PD-1/PD-L1-Inhibitor Monotherapy in Advanced Non-small-Cell Lung Cancer: A Retrospective Bi-centric Cohort Study. Target Oncol. 2019 Dec 1;14(6):707–17.
67. Lefebvre C, Martin E, Hendriks LEL, Veillon R, Puisset F, Mezquita L, et al. Immune checkpoint inhibitors versus second line chemotherapy for patients with lung cancer refractory to first line chemotherapy. Respir Med Res. 2020 Nov 1;78.
68. Li Y, Yang P, Zhou X, Yang X, Wu S. Programmed cell death 1 inhibitor plus chemotherapy vs. chemotherapy in advanced drive-gene-negative non-small-cell lung cancer patients: A real-world study. Front Surg. 2022 Sep 1;9.
69. Liao J, Liu C, Long Q, Wu X, Wang H, Yu H, et al. Direct Comparison Between the Addition of Pembrolizumab or Bevacizumab for Chemotherapy-Based First-Line Treatment of Advanced Non-Squamous Non-Small Cell Lung Cancer Lacking Driver Mutations. Front Oncol. 2021 Sep 29;11.
70. Liao G, Qian Y, Arooj S, Zhao Z, Yan M, Li Z, et al. Radiation Plus Anti-PD-1 Therapy for NSCLC Brain Metastases: A Retrospective Study. Front Oncol. 2021 Oct 21;11.
71. Liu Y, Gao Y, Wang Y, Zhao C, Zhang Z, Li B, et al. A single center analysis of first-line treatment in advanced KRAS mutant non-small cell lung cancer: real-world practice. BMC Cancer. 2022 Dec 1;22(1).
72. Long Y, Xiong Q, Song Q, Li Y, Li X, Qin B, et al. Immunotherapy plus chemotherapy showed superior clinical benefit to chemotherapy alone in advanced NSCLC patients after progression on osimertinib. Thorac Cancer. 2022 Feb 1;13(3):394–403.
73. Lu Z, Ye M, Sun T, Wu S, Lin Z, Zhang X, et al. Pembrolizumab for the better treatment of EGFR-mutant T790M-negative advanced lung adenocarcinoma patients than dual treatment of pemetrexed plus platinum after tyrosine kinase inhibitor treatment failure. Ann Palliat Med. 2022 Jun 1;11(6):2100–9.
74. Mao S, Zhou F, Liu Y, Yang S, Chen B, Xu J, et al. ICI plus chemotherapy prolonged survival over ICI alone in patients with previously treated advanced NSCLC. Cancer Immunology, Immunotherapy. 2022 Jan 1;71(1):219–28.
75. Matsumoto H, Kobayashi N, Somekawa K, Fukuda N, Kaneko A, Kamimaki C, et al. Pembrolizumab monotherapy versus pembrolizumab plus chemotherapy in patients with non-small-cell lung cancer: A multicenter retrospective trial. Thorac Cancer. 2022;13(2):228–35.
76. Miao K, Zhang X, Wang H, Si X, Ni J, Zhong W, et al. Real-World Data of Different Immune Checkpoint Inhibitors for Non-Small Cell Lung Cancer in China. Front Oncol. 2022 Mar 15;12.
77. Miura Y, Mouri A, Kaira K, Yamagguchi O, Shiono A, Hashimoto K, Nishihara F, Shinomiya S, Akagami T, Murayama, Y, Abe, T, Noda S, Kato S Kobayashi K KH. Thoracic Cancer - 2020 - Miura - Chemoradiotherapy followed by durvalumab in patients with unresectable advanced non‐small.pdf. Thorac Cancer; 2020. p. 1280–7.
78. Molinier O, Besse B, Barlesi F, Audigier-Valette C, Friard S, Monnet I, et al. IFCT-1502 CLINIVO: real-world evidence of long-term survival with nivolumab in a nationwide cohort of patients with advanced non-small-cell lung cancer. ESMO Open. 2021 Feb [cited 2021 Dec 27];7(1):100353.
79. Morimoto K, Yamada T, Yokoi T, Kijima T, Goto Y, Nakao A, et al. Clinical impact of pembrolizumab combined with chemotherapy in elderly patients with advanced non-small-cell lung cancer. Lung Cancer. 2021 Nov 1;161:26–33.
80. Morimoto K, Uchino J, Yokoi T, Kijima T, Goto Y, Nakao A, et al. Early discontinuation of induction therapy in chemoimmunotherapy as an effective alternative to the standard regimen in patients with non-small cell lung cancer: a retrospective study. J Cancer Res Clin Oncol.2022 Sep;148(9):2437-2446.
81. Mouritzen MT, Junker KF, Carus A, Ladekarl M, Meldgaard P, Nielsen AWM, et al. Clinical features affecting efficacy of immune checkpoint inhibitors in pretreated patients with advanced NSCLC: a Danish nationwide real-world study. Acta Oncol (Madr). 2022;61(4):409–16.
82. Murteira R, Borges FC, Mendes GP, Ramos C, Ramos A, Soares P, et al. Real-world effectiveness of pembrolizumab in previously treated non-small cell lung cancer: A population-based cohort study. Pharmacoepidemiol Drug Saf. 2020 Oct 1;29(10):1295–302.
83. Nakamura Y, Miyazaki K, Aiko N, Misumi Y, Agemi Y, Taniguchi Y, et al. Efficacy of PD-1 inhibitors in older non-small cell lung cancer patients. Anticancer Res. 2020;40(2):923–8.
84. Offin M, Shaverdian N, Rimner A, Lobaugh S, Shepherd AF, Simone CB, et al. Clinical outcomes, local–regional control and the role for metastasis-directed therapies in stage III non-small cell lung cancers treated with chemoradiation and durvalumab. Radiotherapy and Oncology. 2020 Aug 1;149:205–11.
85. Park JH, You GL, Ahn MJ, Kim SW, Hong MH, Han JY, et al. Real-world outcomes of anti-PD1 antibodies in platinum-refractory, PD-L1-positive recurrent and/or metastatic non-small cell lung cancer, and its potential practical predictors: first report from Korean Cancer Study Group LU19-05. J Cancer Res Clin Oncol. 2021 Aug 1;147(8):2459–69.
86. Peng L, Guo J, Kong L, Huang Y, Tang N, Zhang J, et al. Efficacy of immunotherapy in KRAS-mutant advanced NSCLC: A real-world study in a Chinese population. Front Oncol. 2023 Jan 19;12.
87. Pons-Tostivint E, Hulo P, Guardiolle V, Bodot L, Rabeau A, Porte M, et al. Real-world multicentre cohort of first-line pembrolizumab alone or in combination with platinum-based chemotherapy in non-small cell lung cancer PD-L1 ≥ 50%. Cancer Immunology, Immunotherapy. 2023 Jun 1;72(6):1881–90.
88. Qiu L, Zhao X, Shi W, Sun S, Zhang G, Sun Q, et al. Real-world treatment efficacy of anti-programmed death-1 combined with anti-angiogenesis therapy in non-small cell lung cancer patients. Medicine (United States). 2020 Jun 12;99(24):E20545.
89. Qiao M, Zhou F, Hou L, Li X, Zhao C, Jiang T, et al. Efficacy of immune-checkpoint inhibitors in advanced non-small cell lung cancer patients with different metastases. Ann Transl Med. 2021 Jan;9(1):34–34.
90. Ratnayake G, Shanker M, Roberts K, Mason R, Hughes BGM, Lwin Z, et al. Prior or concurrent radiotherapy and nivolumab immunotherapy in non–small cell lung cancer. Asia Pac J Clin Oncol. 2020 Feb 1;16(1):56–62.
91. Rodríguez-Cid JR, Chards SCC, González-Espinoza IR, Garciá-Montes V, Garibay-Diáz JC, Hernández-Flores O, et al. A comparative study of immunotherapy as second-line treatment and beyond in patients with advanced non-small-cell lung carcinoma. Lung Cancer Manag. 2021 Sep 1;10(3).
92. Ruiz-Patiño A, Arrieta O, Cardona AF, Martín C, Raez LE, Zatarain-Barrón ZL, et al. Immunotherapy at any line of treatment improves survival in patients with advanced metastatic non-small cell lung cancer (NSCLC) compared with chemotherapy (Quijote-CLICaP). Thorac Cancer. 2020 Feb 1;11(2):353–61.
93. Saad A, Goldstein J, Appel S, Daher S, Urban D, Onn A, et al. Chemoradiation followed by adjuvant durvalumab in stage III non–small cell lung cancer: Real-world comparison of treatment outcomes to historical controls treated with chemoradiation alone. Thorac Cancer. 2022 Jun 1;13(12):1763–71.
94. Sabatier R, Nicolas E, Paciencia M, Jonville-Béra AP, Madroszyk A, Cecile M, et al. Nivolumab in routine practice for older patients with advanced or metastatic non-small cell lung cancer. J Geriatr Oncol. 2018 Sep 1;9(5):494–500.
95. Samaranayake C, McCaffrey E, Coucher J, Lehman M, Tao Mai G, Murphy M. Radiation therapy for augmenting the efficacy of immunotherapy in advanced non-small cell lung cancer: a case-controlled study. ERJ open res.2020 Mar 9;6(1);189-209
96. Samuel E, Lie G, Balasubramanian A, Hiong A, So Y, Voskoboynik M, et al. Impact of Radiotherapy on the Efficacy and Toxicity of anti-PD-1 Inhibitors in Metastatic NSCLC. Clin Lung Cancer. 2021 May 1;22(3):e425–30.
97. Sankar K, Bryant AK, Strohbehn GW, Zhao L, Elliott D, Moghanaki D, et al. Real World Outcomes versus Clinical Trial Results of Durvalumab Maintenance in Veterans with Stage III Non-Small Cell Lung Cancer. Cancers (Basel). 2022 Feb 1;14(3).
98. Schouten RD, Egberink L, Muller M, de Gooijer CJ, van Werkhoven E, van den Heuvel MM, et al. Nivolumab in pre-treated advanced non-small cell lung cancer: Long term follow up data from the Dutch expanded access program and routine clinical care. Transl Lung Cancer Res. 2020 Oct 1;9(5):1736–48.
99. Shen CI, Chao HS, Shiao TH, Chiang CL, Huang HC, Luo YH, et al. Comparison of the outcome between immunotherapy alone or in combination with chemotherapy in EGFR-mutant non-small cell lung cancer. Sci Rep. 2021 Dec 1;11(1).
100. Sheng J, Li H, Yu X, Yu S, Chen K, Pan G, et al. Efficacy of PD-1/PD-L1 inhibitors in patients with non-small cell lung cancer and brain metastases: A real-world retrospective study in China. Thorac Cancer. 2021 Nov 1;12(22):3019–31.
101. Shepard MJ, Xu Z, Donahue J, Eluvathingal Muttikkal TJ, Cordeiro D, Hansen L, et al. Stereotactic radiosurgery with and without checkpoint inhibition for patients with metastatic non–small cell lung cancer to the brain: a matched cohort study. J Neurosurg. 2020 Sep 1;133(3):685–92.
102. Shi Y, Ji M, Jiang Y, Yin R, Wang Z, Li H, et al. A cohort study of the efficacy and safety of immune checkpoint inhibitors plus anlotinib versus immune checkpoint inhibitors alone as the treatment of advanced non-small cell lung cancer in the real world. Transl Lung Cancer Res. 2022 Jun 1;11(6):1051–68.
103. Stenehjem DD, Lubinga SJ, Gupte-Singh K, Zhang Y, Le TK, Penrod JR, et al. Real-World Effectiveness of Nivolumab Monotherapy After Prior Systemic Therapy in Advanced Non–Small-Cell Lung Cancer in the United States. Clin Lung Cancer. 2021 Jan 1;22(1):e35–47.
104. Sun S, Liu C, Duan C, Yu S, Zhang Q, Xu N, et al. Efficacy and safety of immune checkpoint inhibitors in post-TKI NSCLC patients harboring EGFR mutations. J Cancer Res Clin Oncol. 2023 Jul 1;149(7):2937–49.
105. Takumida H, Horinouchi H, Masuda K, Shinno Y, Okuma Y, Yoshida T, et al. Comparison of time to failure of pembrolizumab plus chemotherapy versus pembrolizumab monotherapy: a consecutive analysis of patients having NSCLC with high PD-L1 expression. Cancer Immunology, Immunotherapy. 2022 Mar 1;71(3):737–46.
106. Taugner J, Käsmann L, Eze C, Tufman A, Reinmuth N, Duell T, et al. Durvalumab after chemoradiotherapy for PD-L1 expressing inoperable stage III NSCLC leads to significant improvement of local-regional control and overall survival in the real-world setting. Cancers (Basel). 2021 Apr 1;13(7).
107. Tian T, Yu M, Yu Y, Wang K, Tian P, Luo Z, et al. Immune checkpoint inhibitor (ICI)-based treatment beyond progression with prior immunotherapy in patients with stage IV non-small cell lung cancer: a retrospective study. Transl Lung Cancer Res. 2022 Jun 1;11(6):1027–37.
108. Tian T, Yu M, Li J, Jiang M, Ma D, Tang S, et al. Front-Line ICI-Based Combination Therapy Post-TKI Resistance May Improve Survival in NSCLC Patients With EGFR Mutation. Front Oncol. 2021 Nov 23;11.
109. Torasawa M, Yoshida T, Yagishita S, Shimoda Y, Shirasawa M, Matsumoto Y, et al. Nivolumab versus pembrolizumab in previously-treated advanced non-small cell lung cancer patients: A propensity-matched real-world analysis. Lung Cancer. 2022 May 1;167:49–57.
110. Trummer A, Bethge A, Dickgreber N, Dittrich I, Golpon H, Hoffknecht P, et al. NSCLC with uncommon EGFR mutations treated with atezolizumab plus bevacizumab and chemotherapy. Lung Cancer. 2022 Dec 1;174:141–5.
111. Tsai JS, Wei SH, Chen CW, Yang SC, Tseng YL, Su PL, et al. Pembrolizumab and Chemotherapy Combination Prolonged Progression-Free Survival in Patients with NSCLC with High PD-L1 Expression and Low Neutrophil-to-Lymphocyte Ratio. Pharmaceuticals. 2022 Nov 1;15(11).
112. Velcheti V, Chandwani S, Chen X, Pietanza MC, Piperdi B, Burke T. Outcomes of first-line pembrolizumab monotherapy for PD-L1-positive (TPS ≥50%) metastatic NSCLC at US oncology practices. Immunotherapy. 2019;11(18):1541–54.
113. Velcheti V, Hu X, Piperdi B, Burke T. Real-world outcomes of first-line pembrolizumab plus pemetrexed-carboplatin for metastatic nonsquamous NSCLC at US oncology practices. Sci Rep. 2021 Dec 1;11(1).9222
114. Vrankar M, Stanic K, Jelercic S, Ciric E, Vodusek AL, But-Hadzic J. Clinical outcomes in stage III non-small cell lung cancer patients treated with durvalumab after sequential or concurrent platinum-based chemoradiotherapy - Single institute experience. Radiol Oncol. 2021 Nov 19;55(4):482–90.
115. Vrdoljak E, Jakopović M, Geczi L, Bogos K, Bošković L, Magri C, et al. Real-World Safety and Efficacy of Nivolumab in Advanced Squamous and Nonsquamous Non-Small-Cell Lung Cancer: A Retrospective Cohort Study in Croatia, Hungary, and Malta. J Oncol. 2020;2020.
116. Wallrabenstein T, Mamot M, Daetwyler E, König D, Rothschild SI. Real-World Data of Combined Immunochemotherapy in Patients With Nonsquamous Advanced NSCLC. A Single-Center Retrospective Study. JTO Clin Res Rep. 2023 May 1;4(5).
117. Wang Y, Nie J, Dai L, Hu W, Zhang J, Chen X, et al. Evaluation of efficacy and toxicity of nivolumab combined with or without docetaxel in patients with advanced NSCLC. Cancer Immunology, Immunotherapy. 2022 Feb 1;71(2):267–76.
118. Wang M, Li J, Xu S, Li Y, Li J, Yu J, et al. Immunotherapy combined with chemotherapy improved clinical outcomes over bevacizumab combined with chemotherapy as first-line therapy in adenocarcinoma patients. Cancer Med. 2023 Mar 1;12(5):5352–63.
119. Wang X, Niu X, An N, Sun Y, Chen Z. Comparative Efficacy and Safety of Immunotherapy Alone and in Combination With Chemotherapy for Advanced Non-small Cell Lung Cancer. Front Oncol. 2021 Mar 18;11.
120. Wang W, Shao L, Xu Y, Song Z, Lou G, Zhang Y, et al. Efficacy and safety of anlotinib with and without EGFR-TKIs or immunotherapy in the treatment of elder patients with non-small-cell lung cancer: a retrospective study. BMC Pulm Med. 2022 Dec 1;22(1).
121. Wang P, Yin T, Zhao K, Yu J, Teng F. Efficacy of single-site radiotherapy plus PD-1 inhibitors vs PD-1 inhibitors for oligometastatic non-small cell lung cancer. J Cancer Res Clin Oncol. 2022 May 1;148(5):1253–61.
122. Wang Y, Zhang T, Wang J, Zhou Z, Liu W, Xiao Z, et al. Induction Immune Checkpoint Inhibitors and Chemotherapy Before Definitive Chemoradiation Therapy for Patients With Bulky Unresectable Stage III Non-Small Cell Lung Cancer. Int J Radiat Oncol Biol Phys. 2023 Jul 1;116(3):590–600.
123. Weis TM, Hough S, Reddy HG, Daignault-Newton S, Kalemkerian GP. Real-world comparison of immune checkpoint inhibitors in non-small cell lung cancer following platinum-based chemotherapy. Journal of Oncology Pharmacy Practice. 2020 Apr 1;26(3):564–71.
124. Wu K, Fu Y, Zeng D, Chen T, Wang C, Jiang J. A retrospective cohort study of sintilimab and pembrolizumab as first-line treatments for advanced non-small cell lung cancer. J Thorac Dis. 2022 Mar 1;14(3):679–88.
125. Xiong Q, Qin B, Xin L, Yang B, Song Q, Wang Y, et al. Real-World Efficacy and Safety of Anlotinib With and Without Immunotherapy in Advanced Non-Small Cell Lung Cancer. Front Oncol. 2021 Jul 29;11.
126. Xu B, Cheng H, Li K, Lv Y, Zeng X, Liu T, et al. Carboplatin and nab-paclitaxel chemotherapy with or without atezolizumab as front-line management for treatment-naïve metastatic nonsquamous non-small cell lung cancer with PD-L1 staining: a retrospective study. J Cancer Res Clin Oncol. 2022 Nov 1;148(11):3029–38.
127. Yang L, Hao X, Hu X, Wang Z, Yang K, Mi Y, et al. Superior efficacy of immunotherapy-based combinations over monotherapy for EGFR-mutant non-small cell lung cancer acquired resistance to EGFR-TKIs. Thorac Cancer. 2020 Dec 1;11(12):3501–9.
128. Yang Z, Chen Y, Wang Y, Hu M, Qian F, Zhang Y, et al. Pembrolizumab Plus Chemotherapy Versus Chemotherapy Monotherapy as a First-Line Treatment in Elderly Patients (≥75 Years Old) With Non-Small-Cell Lung Cancer. Front Immunol. 2022 Feb 14;13.
129. Li Y, Yang P, Zhou X, Yang X, Wu S. Programmed cell death 1 inhibitor plus chemotherapy vs. chemotherapy in advanced drive-gene-negative non-small-cell lung cancer patients: A real-world study. Front Surg. 2022 Sep 1;9.
130. Yu X, Chu X, Wu Y, Zhou J, Zhao J, Zhou F, et al. Favorable clinical outcomes of checkpoint inhibitorbased combinations after progression with immunotherapy in advanced non-small cell lung cancer. Cancer Drug Resistance. 2021;4(3):728–39.
131. Yu X, Li J, Ye L, Zhao J, Xie M, Zhou J, et al. Real-world outcomes of chemo-antiangiogenesis versus chemo-immunotherapy combinations in EGFR-mutant advanced non-small cell lung cancer patients after failure of EGFR-TKI therapy. Transl Lung Cancer Res. 2021 Sep 1;10(9):3782–92.
132. Zayas-Soriano M, Bonete-Sánchez M, Campillo-López J, Marcos-Ribes B, Hernández-Guio A, Aznar-Saliente MT. Clinical efficacy and safety of anti PD-1/PD-L1 antibodies as monotherapy in patients with non-small-cell lung cancer. Farmacia Hospitalaria. 2021 Jan 1;45(1):22–7.
133. Zhai X, Jing X, Li J, Tian Y, Xu S, Wang M, et al. Clinical Outcomes for PD-1 Inhibitor Plus Chemotherapy as Second-Line or Later Therapy Compared to PD-1/PD-L1 Inhibitor Alone in Advanced Non-small-cell Lung Cancer. Front Oncol. 2020 Sep 30;10.
134. Zhang F, Huang D, Li T, Zhang S, Wang J, Zhang Y, et al. Anti-PD-1 therapy plus chemotherapy and/or Bevacizumab as second line or later treatment for patients with advanced non-small cell lung cancer. J Cancer. 2020;11(3):741–9.
135. Zhang J, Wu D, Zhang Z, Long J, Tian G, Wang Y, et al. Pembrolizumab or Bevacizumab Plus Chemotherapy as First-Line Treatment of Advanced Nonsquamous Nonsmall Cell Lung Cancer: A Retrospective Cohort Study. Technol Cancer Res Treat. 2021;20.
136. Zhang T, Yang X, Zhao J, Xia L, Wang Q, Jin R, et al. The Application of Combined Immune Checkpoint Inhibitor Modalities in Previously Treated Non-Small Cell Lung Cancer Patients and the Associations Thereof With the Lung Immune Prognostic Index. Front Oncol. 2021 Jun 4;11.
137. Zhang X, Zeng L, Li Y, Xu Q, Yang H, Lizaso A, et al. Anlotinib combined with PD-1 blockade for the treatment of lung cancer: a real-world retrospective study in China. Cancer Immunology, Immunotherapy. 2021 Sep 1;70(9):2517–28.
138. Zhang W, Zhang C, Yang S, Chen Q, Wang C, Guo Q. Immune checkpoint inhibitors plus anlotinib versus anlotinib alone as third-line treatment in advanced non-small-cell lung cancer: A retrospective study. Future Oncology. 2021 Nov 1;17(31):4091–9.
139. Zhang Y, Zhu T, Wang Q, Wang J, Chen X. Original Article Effects of PD-1 inhibitor combined with anti-angiogenic drugs on efficacy and immune function of non-small cell lung cancer. Am J Transl Res. 2022.15;14(11):8225-8233.
140. Zhao Z, Hu R, Chen Y, Zhou G, Yu S, Feng J. Efficacy and Safety of PD-1 Immune Checkpoint Inhibitors in Locally Advanced and Advanced Non-Small-Cell Lung Cancer Patients with Chronic Infection. Oncol Res Treat. 2022 Jun 1;45(6):366–73.
141. Zhao X, Wu X, Yu H, Wang H, Sun S, Hu Z, et al. Prognostic value of hematologic parameters in advanced non-small cell lung cancer patients receiving anti-PD-1 inhibitors. Front Immunol. 2022 Oct 20;13.
142. Zheng MM, Tu HY, Yang JJ, Zhang XC, Zhou Q, Xu CR, et al. Clinical outcomes of non–small cell lung cancer patients with leptomeningeal metastases after immune checkpoint inhibitor treatments. Eur J Cancer. 2021 Jun 1;150:23–30.
143. Zhou S, Ren F, Meng X. Efficacy of immune checkpoint inhibitor therapy in EGFR mutation-positive patients with NSCLC and brain metastases who have failed EGFR-TKI therapy. Front Immunol. 2022 Sep 27;13.
144. Zhou ZC, Chen KY, Li N, Xie MY, Sheng JM, Fan Y, et al. Real-world utilization of PD-1/PD-L1 inhibitors with palliative radiotherapy in patients with metastatic non-small cell lung cancer. Thorac Cancer. 2022 Aug 1;13(16):2291–300.
